# Plasma p-tau217 as a two-cutoff approach to improve tau-PET screening and trial recruitment in Alzheimer’s disease

**DOI:** 10.1038/s41598-026-66315-5

**Published:** 2026-08-08

**Authors:** Yasaman Abtahi, Nikki Ghadiminia, Hamide Nasiri, Amir Jannesari Ladani, Kiana Naghavi Khanghah, Marjan Shamspour, Delsa Davashi Jamaloei, Fatemeh Sadat Meybodi, Saba Rahimi, Danial Hashemi, Mohammadreza Saleh, Negin Ashoori, Shayan Shakeri

**Affiliations:** 1 Department of English Language and Literature, Allameh Tabataba’i University, Tehran, Iran; 2 School of Psychology, University of Kent, Canterbury, UK; 3 Student Research Committee, School of Medicine, Zanjan University of Medical Sciences, Zanjan, Iran; 4 Department of Clinical Psychology, Islamic Azad University, Isfahan (Khorasgan) Branch, Isfahan, Iran; 5 Department of Medicine, Student Research Committee, Shahid Beheshti University of Medical Sciences, Tehran, Iran; 6 Department of Psychiatry, Shahid Beheshti Hospital, Afzalipour Faculty of Medicine, Kerman University of Medical Sciences, Kerman, Iran; 7 Faculty of Industrial Engineering, Isfahan University of Technology, Isfahan, Iran; 8 Department of Genetics, Faculty of Biological Sciences, Islamic Azad University North Tehran Branch, Tehran, Iran; 9 Faculty of Pharmacy, Charles University, Hradec Králové, Czech Republic; 10 Department of Medicine, Tehran Medical Sciences Branch, Islamic Azad University, Tehran, Iran; 11 Faculty of Medicine, Student Research Committee, Alborz University of Medical Sciences, Karaj, Iran; 12 Department of Medicine, Ardabil Branch, Islamic Azad University, Ardabil, Iran; 13 Department of Medical Genetics, Faculty of Medical Sciences, Tarbiat Modares University, Tehran, Iran

**Keywords:** Plasma p-tau217, Tau-PET, Alzheimer’s disease, Two-cutoff strategy, Blood-based biomarkers, Biomarkers, Medical research, Neurology, Neuroscience

## Abstract

Blood-based biomarkers could broaden access to Alzheimer’s disease diagnostics, but their ability to approximate tau PET, and to address gray-zone results, remains uncertain. We evaluated plasma p-tau217 against tau PET using a harmonized ADNI baseline cohort and an independently derived temporal meta-ROI tau-PET threshold. We included participants with complete baseline plasma biomarkers (p-tau217, Aβ42, Aβ40, NfL, GFAP), tau PET, and demographics, including: cognitively normal = 229, mild cognitive impairment = 149, and Alzheimer’s disease dementia = 41. ROC analyses assessed discrimination of tau-PET positivity overall and by diagnostic group. A two-cutoff strategy was implemented to minimize indeterminate classifications. Across the pooled cohort, p-tau217 demonstrated the strongest ability to discriminate tau-PET status (AUC 0.671, 95% CI 0.597–0.746), outperforming Aβ42, Aβ40, and Aβ42/Aβ40; Though overall accuracy remained moderate and performance differed based on diagnosis, with AUCs of 0.785 for Alzheimer’s disease dementia and lower values for cognitively normal and mild cognitive impairment. The two-cutoff approach substantially reduced intermediate classifications compared with conventional markers. In ADNI, plasma p-tau217 showed the highest discriminative performance among the single blood biomarker for detecting tau-PET positivity, showing moderate accuracy overall and its strongest performance in Alzheimer’s disease dementia. A two-cutoff approach substantially reduces gray-zone classifications, supporting p-tau217 as a primary screening tool before confirmatory imaging. Further work and validation are needed across different groups.

**Supplementary Information:**

The online version contains supplementary material available at https://doi.org/10.1038/s41598-026-66315-5.

## Introduction

Alzheimer’s disease (AD) is increasingly defined not only by clinical symptoms but also by its underlying biology. The ATN framework, capturing amyloid deposition (A), tau pathology (T), and neurodegeneration (N), has advanced diagnosis beyond syndromic criteria and provided a clearer framework for research and clinical trials^1^. At the same time, blood-based biomarkers are rapidly emerging as practical surrogates for PET and CSF measures, enabling earlier and more widely accessible detection. Among key plasma biomarkers, including p-tau217 and related amyloid or neurodegeneration markers, p-tau217 has shown the strongest capacity to reflect tau pathology^2,3^. The 2024 revision of the NIA-AA criteria further formalized the role of blood biomarkers within this framework, recognizing plasma p-tau217 as a core 1 diagnostic marker alongside CSF Aβ42/40 and amyloid PET, and introducing a continuous biological staging model that situates plasma biomarkers within the broader AD continuum^4^.

A recent study has shown that plasma p-tau217, measured using the Lumipulse platform, was strongly correlated with tau PET uptake in temporal meta-regions (*r* = 0.78) and discriminated tau-positive individuals with an area under the curve of approximately 0.94^3^. Furthermore, plasma p-tau217 in individuals with subthreshold tau PET has been shown to predict accelerated future tau accumulation in amyloid-positive individuals, emphasizing its prognostic relevance beyond cross-sectional classification^5^. Another study found a correlation of *r* = 0.61 (*p* < 0.001) between plasma p-tau217 and cortical tau PET SUVR in amyloid-PET positive patients with mild cognitive impairment (MCI) or AD dementia, with particularly robust associations in temporo-parietal and dorsolateral frontal cortices^6^. Building on these external findings, we aimed to replicate and refine these associations using a well-characterized Alzheimer’s Disease Neuroimaging Initiative (ADNI) cohort and an independently derived tau PET threshold. This approach allowed us to test the generalizability of published p-tau217–tau PET associations while addressing unresolved methodological questions, including cutoff determination and gray-zone classification.

Other plasma markers, including Aβ42/40, GFAP, and NfL, also correlate with amyloid or tau burden, but their specificity and diagnostic reliability vary across cohorts and clinical stages^6,7^. The definition of tau PET positivity thresholds and the handling of “gray-zone” or indeterminate plasma values remain underexplored, as does how dual-cutoff (two-threshold) approaches might improve clinical decision making. To address this, we derived a temporal meta-ROI tau-PET threshold of 1.44 SUVR (± 2.5% gray zone) from an independent cognitively normal reference using a mean + 2 SD rule. In this study, using the ADNI cohort with baseline plasma assays (p-tau217, Aβ42, Aβ40, NfL, GFAP) and tau PET imaging, we assessed the discrimination of tau PET positivity by plasma biomarkers, regional correlations between plasma markers and tau PET burden across diagnostic strata, and finally the value of a two-cutoff strategy to reduce indeterminate classifications.

## Methods

### Participants and study design

This cross-sectional analysis used data from the ADNI database (http://adni.loni.usc.edu/), a public–private partnership launched in 2003 under the leadership of Dr. Michael W. Weiner, Principal Investigator. The overarching aim of ADNI is to evaluate whether serial magnetic resonance imaging (MRI), PET, biomarkers, and clinical and neuropsychological assessments can reliably track the progression of MCI and early AD. Participants, aged 55–90 years, underwent multimodal neuroimaging, lumbar punctures, and longitudinal follow-up assessments. Detailed eligibility criteria for the ADNI cohort have been previously reported^2,3,8^.

Key exclusion criteria at enrollment included a Hachinski Ischemic Score greater than 4, current use of unapproved medications or recent changes in permitted treatments, a Geriatric Depression Scale score of 6 or higher, and fewer than six years of education or equivalent occupational attainment.

In this analysis, we included participants classified as cognitively normal (CN), MCI, or AD at baseline. Diagnostic classification followed ADNI criteria, including (i) Mini-Mental State Examination (MMSE) scores of 24–30 for MCI and 20–24 for AD; (ii) a Clinical Dementia Rating (CDR) global score of 0.5 with a memory box score ≥ 0.5 for MCI and a CDR global score of 0.5 or 1 for AD; or (iii) a Logical Memory II subscale score below the age-specific cutoff. All participants also met diagnostic standards established by the National Institute of Neurological and Communicative Disorders and Stroke and the AD and Related Disorders Association^8,9^.

In addition to ADNI-wide eligibility, this study applied further inclusion criteria. We restricted analyses to participants with complete data for plasma biomarkers, tau PET measures, and demographic variables at the baseline time point, yielding a final sample of 230 CN, 149 MCI, and 41 AD participants. Exclusion criteria for this analysis included missing values in any required modality and the presence of statistical outliers. Outliers in continuous variables were identified within diagnostic groups using the interquartile range (IQR) method and recoded as missing values. To reduce bias from incomplete data, variables with more than 20% missing values after this procedure were excluded from subsequent analyses. The resulting cleaned dataset was used for all subsequent statistical evaluations.

### Plasma biomarker assessment

In the ADNI4 study, plasma biomarkers were analyzed approximately every 4 months in batches at the ADNI Biomarker Core. Analyses employed two validated immunoassay platforms: the Lumipulse G1200 chemiluminescent enzyme immunoassay (Fujirebio) for amyloid-β (Aβ42, Aβ40) and phosphorylated tau at threonine 217 (p-tau217), and the Simoa Quanterix HD-X platform (Quanterix) for NfL and GFAP^10,11^.

Each analytical run included internal quality control plasma pools prepared from cognitively normal individuals and from individuals with abnormal plasma Aβ42/Aβ40 and p-tau values. Plasma Aβ42, Aβ40, and p-tau217 were measured in singlicate on the Lumipulse G1200 platform, whereas NfL and GFAP were measured in duplicate on the Quanterix HD-X platform according to the manufacturer’s protocols. Reported run-to-run precision (coefficient of variation) ranged from 5.1 to 5.4% for Aβ42, 3.7–4.2% for Aβ40, 6.2–9.9% for p-tau217, 6.0–10.6% for NfL, and 6.8–9.9% for GFAP, indicating good analytical reproducibility across assay batches. Plasma biomarker concentrations were expressed in pg/mL and calculated from manufacturer-provided calibration curves. Samples were stored at − 80 °C until batched analysis, which was performed approximately every four months according to the ADNI4 Biomarker Core protocol.

### Tau PET acquisition and processing

Tau PET data were obtained using the radiotracer [^18F]flortaucipir (FTP; also referred to as AV1451 in image filenames). Importantly, only FTP PET data were included in this study; no MK6240 or PI-2620 PET data were analyzed, ensuring tracer consistency across all analyses. PET images were downloaded from the LONI database in fully pre-processed format, including frame realignment and averaging, spatial normalization, and smoothing. Data acquired since September 1, 2022 were smoothed to 6 mm full-width half-maximum (FWHM), whereas earlier FTP data were smoothed to 8 mm^12–16^.

SUVRs were intensity-normalized to the inferior cerebellar gray matter reference region using the SUIT template^17^, and transformed back into native space. This reference region was selected to minimize off-target binding effects in the cerebellum and improve cross-subject comparability.

Regional uptake values were computed for bilateral FreeSurfer Desikan–Killiany ROIs and the temporal meta-ROI composite^18^. Partial volume correction (PVC) was performed using the Geometric Transfer Matrix (GTM) method^12,13,19,20^, incorporating both cortical ROIs and regions susceptible to off-target binding. Although hippocampal signal may be influenced by choroid plexus binding, it was retained to preserve anatomical completeness.

To ensure data quality across multiple processing stages, all PET–MRI registrations and FreeSurfer segmentations underwent systematic visual quality control.

Tau PET positivity (T(+)) was initially evaluated using a published threshold of 1.34 SUVR in the temporal meta-ROI^21^, Given variability across pipelines, we additionally derived a cohort-specific threshold using an independent cognitively normal reference sample processed identically in our pipeline. This yielded a cut-off of 1.44 (mean = 1.22, SD = 0.11), with a gray zone defined as 1.41–1.48 (Supplementary Methods; Supplementary Fig. 1).

### Statistical analysis

All analyses were conducted in Python (pandas v2.1.4, statsmodels v0.14.0, pingouin v0.5.4, SciPy v1.12.0, and numpy v1.26.4). The distribution of continuous variables was assessed using the Shapiro–Wilk test. Normally distributed variables are presented as mean (SD) and categorical variables as counts (n, %). Between-group comparisons were conducted using one-way analysis of variance (ANOVA) for normally distributed variables, Kruskal–Wallis tests for non-normally distributed variables, and chi-square (χ^2^) tests for categorical variables. Within diagnostic groups, general linear regression models were used to examine associations between plasma biomarkers and tau PET measures, adjusting for age, education, and sex. Model assumptions were evaluated by visual inspection of Q–Q plots and residual-versus-fitted plots, with normality tested using Shapiro–Wilk test and multicollinearity assessed by variance inflation factors. Indirect effects were estimated using a non-parametric bootstrap procedure with 5000 resamples. Statistical significance was set at two-sided *p* < 0.05. To account for multiple testing, p-values from regression models were adjusted using the false discovery rate (FDR) method with the Benjamini–Hochberg procedure.

## Results

### Baseline demographics

Baseline demographic and clinical characteristics of the study population are presented in Table 1. Age differed across diagnostic groups, with participants in the AD group being older than those in the CN and MCI groups (*p* < 0.001). The proportion of female participants decreased with increasing disease severity (*p* = 0.002). Educational attainment was higher in the CN group compared with MCI and AD; differences between CN and both patient groups were significant (*p* < 0.001), whereas MCI and AD did not differ (*p* = 0.295). Cognitive performance, as measured by CDR-SB and MMSE, showed a progressive decline across the diagnostic spectrum (all pairwise comparisons *p* < 0.001) (Table 1).

**Table 1:** Baseline demographic characteristics of study population.

		CN (*N* = 229)	MCI (*N* = 149)	AD (*N* = 41)	Comparisons	P value	Overall p value
Age, year		69.51 ± 6.58	71.83 ± 7.65	75.48 ± 7.59	CN vs. MCI	0.003	< 0.001^a^
					CN vs. AD	< 0.001	
					MCI vs. AD	0.008	
Gender	Female	143 (62.4)	70 (47)	17 (41.5)			0.002^b^
	Male	86 (37.6)	79 (53)	24 (58.5)			
Education, year		16.93 ± 1.93	15.87 ± 2.44	15.49 ± 2.16	CN vs. MCI	< 0.001	< 0.001^c^
					CN vs. AD	< 0.001	
					MCI vs. AD	0.295	
CDR-SB		0 ± 0	1.55 ± 1.11	4.05 ± 1.27	CN vs. MCI	< 0.001	< 0.001^c^
					CN vs. AD	< 0.001	
					MCI vs. AD	< 0.001	
MMSE		29.39 ± 0.74	27.66 ± 2.01	23 ± 2.18	CN vs. MCI	< 0.001	< 0.001^c^
					CN vs. AD	< 0.001	
					MCI vs. AD	< 0.001	

CN, Cognitively normal; MCI, Mild cognitive impairment; AD, Alzheimer’s disease; CDR-SB, Clinical dementia rating scale sum of boxes; MMSE, Mini-mental state examination. ^a^ANOVA test. ^b^Chi square test. ^c^Kruskal–Wallis test. Continuous variables are presented with mean ± SD. Categorical variables are presented as numbers (frequencies). P values are significant as 0.05 level.

### Plasma biomarker comparisons

P-tau217 levels increased progressively from CN to MCI and AD (*p* < 0.001). Plasma Aβ42 levels were similar across groups, whereas Aβ40 showed a modest increase with disease severity, with differences between CN and AD (*p* = 0.008) and across groups overall (*p* = 0.001). The Aβ42/Aβ40 ratio decreased progressively from CN to MCI and AD (all pairwise comparisons *p* < 0.001). The p-tau217/Aβ42 ratio increased across groups (*p* < 0.001).

NfL and GFAP also increased with diagnostic severity (both *p* < 0.001) (Table 2).

**Table 2:** Plasma biomarker concentrations across diagnostic groups at baseline.

	CN (*N* = 229)	MCI (*N* = 149)	AD (*N* = 41)	Comparisons	P value	Overall p value
p-tau217	0.11 ± 0.05	0.26 ± 0.23	0.57 ± 0.31	CN vs. MCI	< 0.001	< 0.001^a^
				CN vs. AD	< 0.001	
				MCI vs. AD	< 0.001	
Aβ42	26.35 ± 4.78	25.40 ± 5.09	25.72 ± 5.05	CN vs. MCI	0.108	0.223^a^
				CN vs. AD	0.314	
				MCI vs. AD	0.931	
Aβ40	287.05 ± 43.69	295.59 ± 49.02	316.61 ± 64.38	CN vs. MCI	0.088	0.001^b^
				CN vs. AD	0.008	
				MCI vs. AD	0.06	
Aβ42/Aβ40	0.09 ± 0.01	0.09 ± 0.01	0.08 ± 0.01	CN vs. MCI	< 0.001	< 0.001^a^
				CN vs. AD	< 0.001	
				MCI vs. AD	0.002	
p-tau217/Aβ42	0 ± 0	0.01 ± 0.01	0.02 ± 0.02	CN vs. MCI	< 0.001	< 0.001^a^
				CN vs. AD	< 0.001	
				MCI vs. AD	< 0.001	
NfL	15.80 ± 6	20.81 ± 8.78	25.63 ± 7.39	CN vs. MCI	< 0.001	< 0.001^a^
				CN vs. AD	< 0.001	
				MCI vs. AD	0.001	
GFAP	129.4 ± 56.73	151.31 ± 65.85	229.54 ± 78.75	CN vs. MCI	0.002	< 0.001^a^
				CN vs. AD	< 0.001	
				MCI vs. AD	< 0.001	

CN, cognitively normal; MCI, mild cognitive impairment; AD, Alzheimer’s disease; p-tau217, plasma phosphorylated tau at threonine 217; Aβ42, plasma amyloid-β 42; Aβ40, plasma amyloid-β 40; NfL, neurofilament light chain; GFAP, glial fibrillary acidic protein. ^a^Kruskal–Wallis test. ^b^ANOVA test. Continuous variables are presented with Mean ± SD. P values are significant as 0.05 level.

### Associations between plasma biomarkers and regional tau PET uptake

Plasma biomarkers showed statistical associations with regional tau PET uptake that varied across diagnostic groups (Tables S1–S7).

### AD group

In the AD group, lower plasma Aβ42 levels were associated with greater regional tau PET uptake, predominantly within frontal, posterior cingulate, and medial temporal regions (Table 3). Similarly, lower Aβ42/Aβ40 ratios were associated with higher tau burden in limbic structures, including the hippocampus and entorhinal cortex. NfL demonstrated positive associations primarily within limbic regions, whereas p-tau217 exhibited the strongest and most widespread associations, encompassing temporal, limbic, and medial temporal cortices. The p-tau217/Aβ42 ratio showed a similar but slightly less extensive regional pattern (Table 3).

**Table 3:** Significant associations between plasma biomarkers and tau PET findings in AD group.

ROIs	Standardized β	t-value	F-value	Adjusted *R*²	95% CI		*P*-Value
					Upper	Lower	
Aβ42							
ctx-lh-caudalmiddlefrontal	− 0.41	− 2.61	4.06	0.27	− 0.07	− 0.01	0.014
ctx-lh-superiorfrontal	− 0.47	− 2.46	2.03	0.11	− 0.04	0.00	0.020
ctx-bilateral-parahippocampal	− 0.41	− 2.73	4.21	0.26	− 0.07	− 0.01	0.041
ctx-bilateral-posteriorcingulate	− 0.45	− 2.57	3.49	0.24	− 0.07	− 0.01	0.016
ctx-bilateral-precuneus	− 0.46	− 2.58	2.71	0.18	− 0.05	− 0.01	0.032
ctx-rh-parahippocampal	− 0.42	− 2.71	3.58	0.22	− 0.08	− 0.01	0.042
Aβ42/Aβ40							
ctx-entorhinal	− 0.38	− 2.57	4.10	0.25	− 56.32	− 6.60	0.034
ctx-rh-entorhinal	− 0.37	− 2.52	3.79	0.23	− 61.14	− 6.55	0.039
ctx-lh-entorhinal	− 0.37	− 2.60	4.61	0.28	− 56.05	− 6.87	0.027
Bilateral-hippocampus	− 0.43	− 2.79	2.86	0.16	− 32.86	− 5.18	0.027
Left-hippocampus	− 0.43	− 2.79	2.89	0.17	− 34.04	− 5.33	0.030
Right-hippocampus	− 0.40	− 2.59	2.55	0.14	− 32.51	− 3.90	0.026
NfL							
Bilateral-amygdala	0.49	2.74	2.92	0.17	0.01	0.07	0.049
Bilateral-hippocampus	0.46	2.55	2.24	0.12	0.00	0.04	0.027
Left-amygdala	0.56	3.24	3.78	0.23	0.02	0.08	0.013
Left-hippocampus	0.43	2.38	2.10	0.10	0.00	0.04	0.040
Right-hippocampus	0.47	2.57	2.21	0.11	0.00	0.04	0.026
tau217							
ctx-bilateral-entorhinal	0.56	4.13	7.57	0.40	0.56	1.66	0.002
ctx-lh-entorhinal	0.50	3.65	7.09	0.38	0.44	1.56	0.005
ctx-rh-entorhinal	0.57	4.19	7.46	0.40	0.63	1.82	0.001
ctx-rh-middletemporal	0.71	5.77	10.39	0.51	0.98	2.05	< 0.001
ctx-bilateral-temporalpole	0.56	4.45	10.09	0.49	0.41	1.10	0.001
Bilateral-hippocampus	0.60	4.08	5.16	0.30	0.32	0.94	0.002
Left-hippocampus	0.57	3.81	4.74	0.28	0.29	0.95	0.004
Right-hippocampus	0.60	4.09	5.05	0.29	0.32	0.96	0.002
tau217/Aβ40							
bilateral-amygdala	0.43	2.52	2.80	0.15	2.98	27.59	0.049
ctx-bilateral-entorhinal	0.47	3.06	5.25	0.30	6.05	29.85	0.015
ctx-lh-entorhinal	0.43	2.78	5.24	0.30	4.47	28.52	0.026
ctx-lh-temporalpole	0.59	4.00	6.37	0.36	6.62	20.30	0.002
ctx-bilateral-temporalpole	0.54	3.97	8.79	0.44	6.90	21.34	0.001
Bilateral-hippocampus	0.44	2.56	2.42	0.12	1.83	15.92	0.027
Left-hippocampus	0.42	2.44	2.37	0.12	1.47	16.12	0.040
Right-amygdala	0.45	2.66	2.96	0.16	3.74	27.85	0.041
Right-hippocampus	0.44	2.56	2.35	0.12	1.90	16.30	0.026

### MCI and CN groups

In participants with MCI, p-tau217 and p-tau217/Aβ42 exhibited widespread positive associations with regional tau PET uptake, whereas GFAP showed more limited associations that were largely confined to frontal regions (Tables 4 and 5). In cognitively normal individuals, significant associations were sparse and primarily limited to an inverse relationship between the Aβ42/Aβ40 ratio and bilateral amygdala tau uptake (Tables S1–S7).

**Table 4:** Significant associations between plasma biomarkers and tau PET findings in CN.

ROIs	Standardized β	t-value	F-value	Adjusted *R*²	95% CI		*P*-Value
					Upper	Lower	
Aβ42/Aβ40							
Bilateral-amygdala	− 0.22	− 3.17	5.15	0.08	− 4.42	− 1.03	0.011
Left-amygdala	− 0.21	− 3.06	4.20	0.06	− 4.80	− 1.03	0.015
Right-amygdala	− 0.19	− 2.84	4.98	0.07	− 4.18	− 0.76	0.030

**Table 5:** Significant associations between plasma biomarkers and tau PET findings in MCI group.

ROIs	Standardized β	t-value	F-value	Adjusted *R*²	95% CI		*P*-Value
					Upper	Lower	
GFAP							
ctx-lh-soperiorfrontal	0.32	3.38	4.81	0.11	0.00	0.00	0.001
ctx-rh-frontalpole	0.20	2.19	3.84	0.08	0.00	0.00	0.042
tau217							
ctx-bilateral-frontalpole	0.33	3.98	6.20	0.14	0.11	0.34	0.001
ctx-rh-frontalpole	0.35	4.31	7.54	0.16	0.14	0.39	< 0.001
tau217/Aβ40							
ctx-lh-frontalpole	0.28	3.19	3.45	0.07	1.80	7.65	0.012

**Fig. 1:**
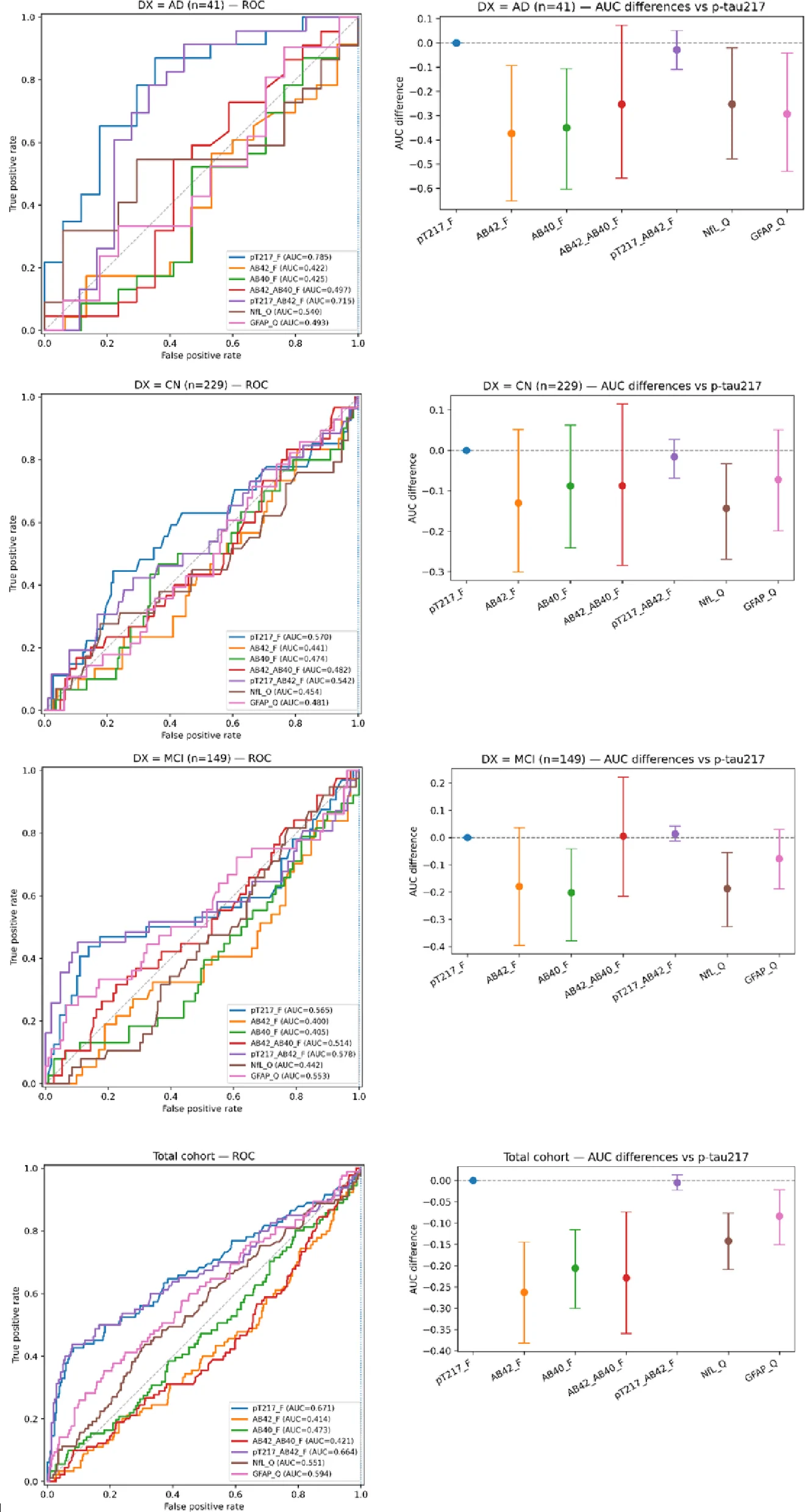
Left: plasma biomarkers receiver operating characteristic (ROC) curves for predicting tau PET positivity in AD, CN, and MCI groups. Right: Plots represent the difference in area under the curve (AUC) between each biomarker and p-tau217 (reference set to 0.0). AUC values closer to 1.0 indicate better diagnostic discrimination, whereas values around 0.5 indicate performance no better than chance. Positive values in the right panel indicate better performance of p-tau217 compared with the respective biomarker, while negative values indicate superior performance of the comparator biomarker. AD, Alzheimer’s disease; CN, cognitively normal; MCI, mild cognitive impairment.

### ROC analysis of plasma biomarkers for tau PET positivity

In CN individuals (*n* = 229), p-tau217 showed limited discriminative performance for tau PET positivity (AUC  0.570, 95% CI 0.446–0.702), while Aβ42 (AUC 0.441), Aβ40 (AUC 0.474), GFAP (AUC  0.481), and NfL (AUC 0.454) performed similarly. NfL reached nominal significance (*p* = 0.018) but did not survive FDR correction (Fig. 1).

In MCI, p-tau217 again showed modest performance (AUC 0.565, 95% CI 0.437–0.691). Aβ42 and Aβ40 showed lower performance, with Aβ40 remaining significant after FDR correction. NfL showed weak but statistically detectable discrimination after correction, while GFAP was not significant.

In the pooled cohort, p-tau217 showed the highest discriminative performance (AUC 0.671, 95% CI 0.597–0.746). Aβ42, Aβ40, and Aβ42/Aβ40 ratio showed significantly lower performance (*p* < 0.01, FDR-adjusted *p* < 0.05). NfL and GFAP showed moderate discriminative ability, both reaching significance after correction. The p-tau217/Aβ42 ratio provided only marginal improvement over p-tau217 alone, with largely comparable overall diagnostic performance.

**Fig. 2:**
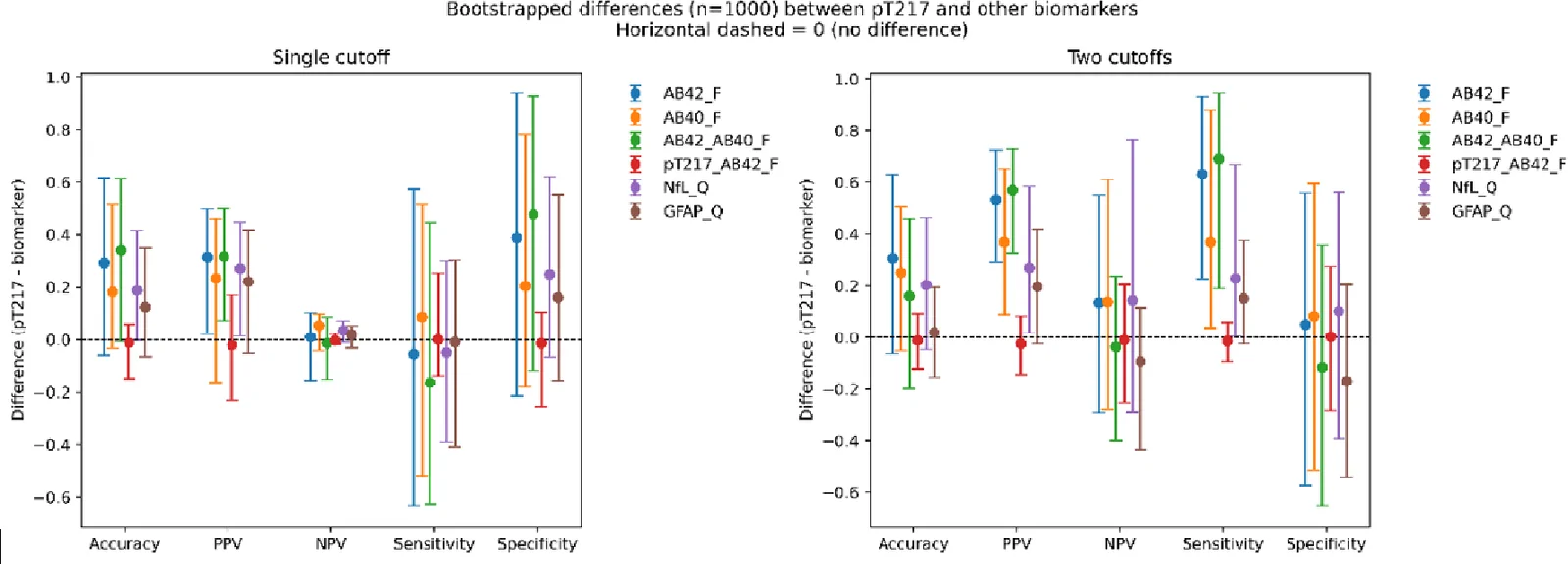
Comparative analysis of diagnostic performance metrics between p-tau217 and plasma biomarkers. Values above the horizontal dashed line (0) indicate superior performance by p-tau217, while values below indicate superior performance by the comparator biomarker. Performance differences were calculated using a single-threshold approach (left) and a dual-threshold approach (right), where the dual-threshold method introduces an intermediate category to account for uncertainty near classification cutoffs. In the dual-threshold approach, individuals are classified into “clearly normal,” “clearly abnormal,” and “intermediate” categories, with the intermediate zone representing values near the decision boundary where classification uncertainty is highest. AD, Alzheimer’s disease; CN, cognitively normal; MCI, mild cognitive impairment.

### Performance of plasma biomarkers in predicting tau positivity using the two-cutoff approach

Diagnostic performance metrics for all plasma biomarkers are presented in Fig. 2 and Table S-8. In the total cohort, plasma p-tau217 demonstrated the highest diagnostic performance among individual biomarkers, achieving an accuracy of 0.81, PPV of 0.58, NPV of 0.85, sensitivity of 0.42, and specificity of 0.92. The p-tau217/Aβ42 ratio further improved overall performance, yielding the highest accuracy (0.82), PPV (0.59), NPV (0.86), sensitivity (0.44), and maintaining a specificity of 0.92.

Across diagnostic subgroups, p-tau217 consistently outperformed the remaining individual biomarkers. CN participants, p-tau217 achieved an accuracy of 0.74, sensitivity of 0.45, and specificity of 0.78, whereas Aβ42 showed the highest specificity (0.96) but poor sensitivity (0.07). Among participants with MCI, p-tau217 demonstrated an accuracy of 0.77, PPV of 0.48, NPV of 0.84, sensitivity of 0.44, and specificity of 0.86, while the p-tau217/Aβ42 ratio further improved diagnostic accuracy to 0.80 and specificity to 0.90. In the AD subgroup, p-tau217 remained the best-performing individual biomarker, with an accuracy of 0.78, PPV of 0.77, NPV of 0.79, sensitivity of 0.87, and specificity of 0.65. The p-tau217/Aβ42 ratio achieved the highest sensitivity (0.91) while maintaining a comparable overall accuracy (0.76).

By comparison, Aβ42, Aβ40, NfL, and GFAP exhibited less balanced diagnostic performance, typically demonstrating either high specificity at the expense of sensitivity or vice versa. Overall, p-tau217-based biomarkers provided the most consistent discrimination across the AD continuum, with modest additional improvement observed when combined with plasma Aβ42.

## Discussion

In this ADNI-based sample, we found that plasma p-tau217 was the strongest single biomarker for discriminating tau PET positivity across the diagnostic spectrum (CN, MCI, AD dementia), consistent with findings from Feizpour et al. (2024) and Mundada et al. (2023). Although plasma p-tau217 was the highest-performing single biomarker in our cohort (pooled AUC 0.671; AD subgroup 0.785; CN 0.570; MCI 0.565), its discriminative ability was only moderate and lower than that reported in some external studies. Variations in PET tracer choice, assay platform, cutoff definition, and case mix across disease stages are plausible contributors to this discrepancy and need to be considered when making cross-cohort comparisons. In contrast, Feizpour et al. reported markedly stronger performance, with plasma p-tau217 measured on the Lumipulse platform correlating with temporal meta-region tau PET uptake (*r* = 0.78) and achieving an AUC of approximately 0.94 for identifying tau-positive individuals^22^. Mundada et al. reported a correlation of *r* = 0.61 between plasma p-tau217 and cortical FTP-SUVR, particularly in temporo-parietal and dorsolateral frontal regions, in amyloid-positive MCI/AD dementia patients^6^. Plausible contributors to this discrepancy include (A) Cohort composition: the ADNI cohort includes a large proportion of CN and MCI participants with presumably lower and more heterogeneous tau burden, which dilutes overall AUC; (B) Variations in PET tracer choice: FTP, MK-6240, and PI-2620 comparison without a harmonized cross-tracer SUVR space and standard normalization, introduces variance between studies; our study exclusively used [^18F] FTP PET with a strict temporal meta-ROI–based SUVR threshold for tau positivity, which may yield more conservative classification performance compared with broader or composite tau PET definitions used elsewhere. (C) cutoff definition: the use of 1.44 SUVR cut-off in our study may differ systematically from those in comparator studies, affecting the proportion of positives and negatives in the ROC analysis.

**Fig. 3:**
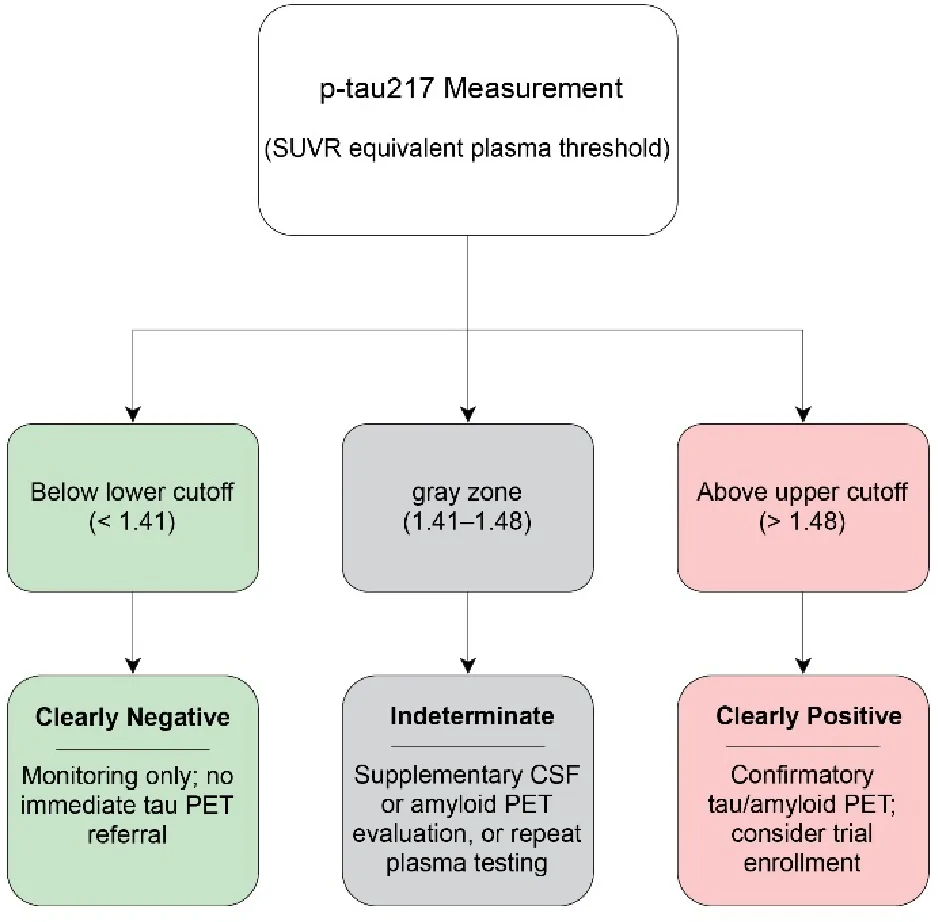
Proposed two-cutoff clinical workflow for plasma p-tau217 as a first-line triage tool. Individuals are classified as clearly negative (below SUVR 1.41), indeterminate (gray zone 1.41–1.48), or clearly positive (above SUVR 1.48), based on a temporal meta-ROI threshold of 1.44 derived from a cognitively normal reference sample (± 2.5% gray zone).

Using dual thresholds, p-tau217 and p-tau217/Aβ42 yielded the lowest intermediate fractions across strata (CN 8–10%; MCI 9–11%; AD 6–8%), whereas Aβ42, Aβ40, and NfL exhibited substantially higher intermediate rates (range 40–65%). The dual-threshold approach reduced the proportion of indeterminate cases for both p-tau217 and the p-tau217/Aβ42 ratio, enhancing clarity for clinical interpretation. These dataset-specific values support the use of p-tau217-based rules as a front-end triage tool. Individuals classified as clearly negative could be just monitored without immediate tau PET referral. For those who classified as clearly positive the confirmatory tau or amyloid PET could be considered. Individuals falling in the gray zone could be referred for supplementary evaluations such as CSF tau or amyloid PET or scheduled for repeat plasma testing after an interval. The proposed triage pathway based on this two-cutoff framework is summarized in Fig. 3. This method would be especially effective in trial-screening area, reducing the number of tau PET scans needed to identify eligible participants and helping the logistics by lowering trial costs.

These associations align with existing literature. plasma p-tau217 strongly correlates with tau PET measures in both symptomatic and preclinical phases^6,22^, and outperforming or matching other plasma markers in reflecting tau accumulation. GFAP and NfL provide useful complementary information (e.g., on neurodegeneration or astroglial response) but do not match the specificity of p-tau217 for tau PET positivity^3,7^. Notably, soluble p-tau217 has been shown to reflect both amyloid and tau pathology simultaneously and to statistically mediate the association between amyloid burden and downstream tau accumulation, providing a mechanistic basis for its performance as a tau PET potential replacement^23^. However, the diagnostic performance metrics observed in the present study are lower than those reported in published ADNI-based and multi-cohort studies. A study reported AUCs of 0.93–0.97 for tau PET detection using the ALZpath pTau217 immunoassay^24^, and a meta-analysis confirmed pooled sensitivity and specificity of 83% for plasma p-tau217 against tau PET positivity across 30 studies^25^, both substantially exceeding the pooled AUC of 0.671, sensitivity of 72.4%, and PPV of 38.7% observed here. Other studies similarly reported strong tau PET prediction in ADNI samples restricted to amyloid-positive participants, a selection criterion not applied in the present study^26,27^. This distinction is the principal explanatory factor. The inclusion of a large proportion of amyloid-negative CN participants in our unselected cohort reduces tau PET positivity prevalence, which directly attenuates PPV through Bayesian dilution, and simultaneously lowers AUC by introducing a subgroup which in plasma p-tau217 has inherently limited discriminative capacity against tau PET. These considerations do not undermine the two-cutoff framework proposed here, which is proposed precisely to manage diagnostic uncertainty in heterogeneous, unselected populations.

Regional tau PET analyses across diagnostic groups revealed a spatial distribution consistent with the canonical trajectory of AD-related tau spread. In CN older adults, tau PET binding is elevated primarily in the medial temporal lobes, consistent with Braak I/II regions observed in autopsy studies. Among MCI participants, involvement extended beyond medial temporal structures, with tau signal spreading into the lateral temporal and posterior cingulate cortices. In AD dementia, progression is characterized by increased intensity and spread of tau binding to parietal and other neocortical regions^28^. This distribution closely mirrors the established Braak staging framework. Tau pathology originates in the transentorhinal cortex, spreads to limbic and temporal areas, then extends into isocortical association areas, and finally reaches unimodal sensory and motor cortex. In vivo tau PET studies have recapitulated this hierarchical pattern, with early stages marked by entorhinal and hippocampal involvement, intermediate stages by neocortical extension into frontal and insular cortices, and later stages by impairment of temporal, parietal, and primary sensorimotor regions^29^. Although the present cross-sectional design precludes direct inference of temporal propagation, more than 80% of amyloid-beta positive participants across diagnostic groups follow typical Braak staging, supporting the interpretation that the observed gradient reflects the expected hierarchical involvement of brain regions across the AD continuum rather than a novel spatial staging pattern.

The clinical implications are meaningful. In cognitively impaired patients, plasma p-tau217 measured on automated immunoassay platforms has demonstrated AUCs of 0.93–0.96 against CSF-defined AD pathology^30^. Additionally, plasma p-tau217 has been shown to achieve diagnostic accuracy near equivalent to clinically used CSF tau217 assays for amyloid PET classification, while remaining less sensitive than CSF in discriminating tau PET positivity^31^. This is consistent with evidence that tau PET and plasma p-tau biomarkers do not measure identical biological constructs. Tau PET captures insoluble neurofibrillary tangle burden whereas plasma p-tau217 primarily reflects soluble phosphorylated tau species, which show differential associations with amyloid load, cognitive decline, and neurodegeneration^32^. These indicate while amyloid PET and CSF biomarkers hold their status as reference standards for definitive confirmation^4^, plasma p-tau217 (and ratios like p-tau217/Aβ42) shows promise as a first-line triage tool within a tiered diagnostic framework, though the moderate discriminative performance observed in this cohort underscores that confirmatory imaging or CSF assessment remains essential before definitive clinical decisions. Two-cutoff strategies, such as the one in the present study, enhance this triage function by reducing the number of indeterminate cases and routing them to confirmatory PET or CSF assessment: strong positives might warrant immediate confirmatory imaging, while intermediate or gray-zone results would prompt follow-up assessments or supplementary biomarkers. In clinical trials, p-tau217 may help enrich samples with participants more likely to exhibit elevated tau PET burden. Recent regulatory progress reinforces translation: Feizpour et al. noted that the Lumipulse pTau217 assay is approaching clinical implementation, and other assays are currently under FDA evaluation for amyloid- and tau-based blood diagnostics^6,22^.

It should be noted that the plasma biomarker panel evaluated in the present study reflects assays available and standardized within ADNI4 at the time of data collection, and does not include more recently developed markers that have demonstrated superior performance for tau PET detection. Specifically, eMTBR-tau243, a microtubule-binding region fragment of tau, and %p-tau217, the ratio of phosphorylated to total tau at threonine 217, have both been reported to identify tau PET SUVR with higher sensitivity, PPV, and NPV than conventional p-tau217 assays^33–35^. The two-cutoff framework proposed in the present study is conceptually adaptable to these next-generation markers and may yield even lower intermediate classification rates when applied to eMTBR-tau243 or %p-tau217. Future work should systematically evaluate whether the two-cutoff strategy provides additive value when applied to these superior biomarkers within ADNI or equivalent cohorts.

Limitations include the cross-sectional design, which precludes establishing temporal causality (e.g., whether increases in plasma p-tau217 precede regional tau PET accumulation). Despite being well characterized, the ADNI cohort is comparatively less diverse in terms of ethnicity, comorbidities, and socioeconomic background, potentially limiting the generalizability of findings to broader clinical populations. ADNI participants are predominantly non-Hispanic White individuals with above average educational attainment and relatively low comorbidity burden. Underrepresented groups comprise only approximately 11% of enrolled participants which is substantially lower than their representation in the US population of adults^36^. This may alter the optimal placement of diagnostic thresholds, as cutpoint values vary across cohorts of different ancestry and geographic origin. Additionally, the AD subgroup relatively small size provides limited statistical power for subgroup-specific ROC analyses and regression models, as reflected in the wide 95% confidence intervals observed. These estimates should be interpreted with appropriate caution and that replication in a larger AD sample is warranted before drawing conclusions specific to this diagnostic group. Heterogeneity in tau tracers (FTP, MK-6240, PI-2620) and preprocessing may introduce variability in SUVRs and cut-points despite a unified pipeline, possibly reducing apparent discriminative performance. Assay calibration, PET tracer differences, and threshold selection remain additional sources of variation across sites. The gray-zone margin was set to ± 2.5% SUVR following the convention established in prior work^37^; this value was adopted as a pragmatic and precedent-based choice rather than one derived through statistical optimization for the current cohort, and sensitivity analyses with alternative gray zone widths (e.g., ± 5% or data-driven intervals) would be informative in future studies. Moreover, region-level correlations differ, with some brain regions exhibiting weaker plasma-PET coupling, particularly early in the disease or at low tau burden.

Finally, the present findings are restricted to participants along the AD clinical spectrum and the performance of plasma p-tau217 should not be generalized to non-AD tauopathies. Likewise, the tau PET tracers employed in this study demonstrate differential off-target binding and variable sensitivity across non-AD tauopathies, such that the SUVR threshold of 1.44 derived here from an AD-spectrum reference sample is not directly applicable for other cases. Extending a two-cutoff plasma biomarker framework to non-AD tauopathies would therefore require independent validation in pathologically confirmed cohorts and potentially different biomarker platforms or tracer-specific thresholds.

Longitudinal evidence already demonstrates that plasma p-tau217 and tau PET independently and jointly predict future cognitive decline in cognitively unimpaired individuals, reinforcing the rationale for early pre-symptomatic screening and supporting the clinical trial enrichment strategy proposed in the present study^32^. Future research should include longitudinal studies using ADNI or preferably other cohorts, tracking both plasma biomarker changes and tau PET accumulation over time to clarify temporal dynamics and improve prognostic accuracy. External validation of the proposed two-cutoff approach in more diverse populations, both ethnically and clinically, will facilitate the development of broadly applicable criteria. Assay harmonization across platforms and tracers is also essential. Future analyses can also formally classify participants by tau PET Braak stage using established regional definitions and evaluate the correspondence with plasma p-tau217 and p-tau217/Aβ42 levels, which was beyond the pre-specified scope of the current study. Finally, implementation studies should examine strategies for handling gray-zone plasma results and evaluate the benefits and limitations of plasma-based screening versus PET confirmation. Emerging deep learning approaches that generate synthetic tau PET scans from plasma biomarkers, MRI, and demographic data represent a complementary methodological direction that could further reduce reliance on confirmatory PET imaging in resource-limited settings^38^.

## Conclusion

In this ADNI-based study, plasma p-tau217 demonstrated the highest discriminative performance among single blood biomarker for identifying tau PET positivity across the AD continuum, with statistically significant advantages over Aβ42, Aβ40, GFAP, and NfL. Although its discriminative accuracy was moderate and somewhat lower than that reported in certain external cohorts, dual-cutoff application substantially reduced gray-zone classifications and improved diagnostic certainty, reinforcing p-tau217’s potential role within a tiered diagnostic framework as a first-line screening tool, pending validation in larger and more diverse cohorts. Regional analyses further confirmed strong and widespread plasma–tau PET correlations, particularly within temporo-parietal and medial temporal regions, while GFAP and NfL provided complementary but less specific information on neurodegeneration.

Our results indicate that p-tau217 and to a marginally greater extent the p-tau217/Aβ42 ratio could help determine which patients should proceed to confirmatory imaging and facilitate the selection of appropriate participants in therapeutic trials. They also emphasize the need for harmonized assay procedures, consistent PET thresholding, and validation in larger and more diverse longitudinal cohorts. Together, our data support the integration of plasma p-tau217–based algorithms into precision diagnostics for AD and provide a framework for scalable, biomarker-driven clinical decision-making.

## Supplementary Information

Below is the link to the electronic supplementary material.


Supplementary Material 1


## Data Availability

Data used in the preparation of this article were obtained from the Alzheimer’s Disease Neuroimaging Initiative (ADNI) database (https://adni.loni.usc.edu). The data are publicly available to qualified investigators upon registration and approval through the ADNI Data Sharing and Publications Committee. Investigators can apply for access at (https://adni.loni.usc.edu) .
