# Supplementary material for: Plasma p-tau217 as a two-cutoff approach to improve tau-PET screening and trial recruitment in Alzheimer’s disease
Source: Sci Rep. 2026 Aug 8;16:27086. doi: 10.1038/s41598-026-66315-5 (PMC13524928; doi:10.1038/s41598-026-66315-5)
Supplement: Supplementary file 1 [file 41598_2026_66315_MOESM1_ESM.docx]

**Supplementary Methods**
**Evaluation of Tau PET Threshold Applicability**

To assess the applicability of a previously published tau PET threshold for the temporal meta-ROI (1.34 SUVR) in our cohort, we followed the methodology described by Ossenkoppele et al. (2018). All available tau PET scans from the ADNI dataset were initially considered. To avoid selection bias in participants with multiple scans, only the first scan per individual was retained, yielding 603 unique baseline scans.

From this set, 229 cognitively normal (CN) participants included in our main study cohort were excluded, leaving an independent reference group of 310 CN individuals. Tau PET positivity [T(+)] was then evaluated using the distribution of tau PET SUVR values in the temporal meta-ROI of these 310 CN participants. Outliers were identified as SUVR values below Q1 – 1.5×IQR or above Q3 + 1.5×IQR and excluded (Supplementary Fig. 1A–B). The threshold was subsequently defined as the mean SUVR plus two standard deviations (mean + 2×SD). This approach was selected to maintain consistency with the methodology used by Ossenkoppele et al. (2018), thereby facilitating comparisons across studies. In addition, the mean + 2×SD method is a simple and commonly used strategy for defining abnormal biomarker values in reference populations. Although alternative approaches, including Gaussian mixture modeling, percentile-based methods, and externally validated thresholds, may also be informative, these methods can be influenced by cohort composition and model assumptions.

In our reference group, the mean SUVR was 1.21 with a standard deviation of 0.11, resulting in a calculated tau PET threshold of 1.44. To account for potential measurement variability near the cutoff, we defined an operational uncertainty interval of ±2.5% around the threshold (1.41–1.48), which was used as a pragmatic gray zone rather than a biologically validated boundary. Although this threshold is slightly higher than the previously reported 1.34 (range 1.30–1.36) by Ossenkoppele et al., differences likely reflect cohort-specific characteristics, preprocessing pipelines, and scanner variability. Importantly, both thresholds were derived using the same methodology, supporting the robustness and comparability of this approach across independent datasets.

**Amyloid and tau pathology distribution**

To characterize the biological context of tau PET findings, we examined the distribution of amyloid PET status across diagnostic groups. Amyloid positivity differed significantly across diagnostic categories (χ² = 14.90, p = 0.00058), with the highest prevalence in AD (63.4%), followed by MCI (38.3%) and CN (31.9%), indicating expected enrichment of amyloid pathology with disease severity.

Similarly, tau PET positivity also differed significantly across diagnostic groups (χ² = 39.76, p < 0.001), with increasing prevalence from CN (13.1%) to MCI (25.5%) and AD (56.1%), confirming the expected staging of tau pathology across the Alzheimer’s disease continuum.

Importantly, all primary analyses in this study focus on tau PET–related associations rather than amyloid PET mechanisms; amyloid status was included only as a descriptive and contextual variable.

**Supplementary Figure 1.** Evaluation of the ADNI-based tau PET threshold using the mean + 2×SD method in CN individuals.
A. Distribution of tau PET SUVR values in the temporal meta-ROI across all CN participants.
B. Identification and exclusion of outliers (red triangles), defined as SUVR values < Q1 – 1.5×IQR or > Q3 + 1.5×IQR.
C. Determination of the tau PET positivity threshold after outlier exclusion. The threshold (red line) was calculated as 1.21 + 2×0.11 ≈ 1.44, with a gray zone defined as 1.44 ± 2.5% (1.41–1.48, dashed black lines).

[ADNI = Alzheimer’s Disease Neuroimaging Initiative; SUVR = standardized uptake value ratio; Q1 = first quartile; Q3 = third quartile; IQR = interquartile range; SD = standard deviation]

**Results**

**Performance of Plasma Biomarkers in Predicting Tau Positivity Using the Two-Cutoff Approach**

We applied the two-cutoff approach to evaluate the diagnostic performance of plasma biomarkers by stratifying individuals into three categories: clearly normal, clearly abnormal, and intermediate. Across the total cohort, plasma p-tau217/ Aβ42 demonstrated the lowest percentage of intermediate cases (15.5%), followed by p-tau217 (14.2%) and GFAP (11.8%), indicating stronger discriminatory power compared with Aβ42, Aβ40, and NfL, which all exhibited higher intermediate proportions (>90%).

When stratified by diagnostic groups, AD patients showed the most favorable performance for plasma p-tau217 (25.0% intermediate) and p-tau217/Aβ42 (19.5% intermediate), both outperforming Aβ40, NfL, and GFAP (intermediate >97%). In the CN group, the lowest intermediate percentages were observed for p-tau217/Aβ42 (5.9%) and p-tau217 (5.9%), while Aβ42 and NfL displayed relatively higher proportions (>91%). For MCI, p-tau217/Aβ42 again showed superior performance (13.9%), followed by p-tau217 (12.8%), whereas Aβ42, Aβ40, and NfL reached intermediate percentages exceeding 96%.

Taken together, these findings highlight that plasma p-tau217 and p-tau217/Aβ42 consistently outperform conventional plasma biomarkers, yielding fewer intermediate classifications and providing improved diagnostic certainty across the Alzheimer’s disease continuum.

Associations between plasma Aβ40 and tau PET

In AD participants, higher plasma Aβ40 was significantly associated with lower tau PET uptake in the left caudal middle frontal cortex (β = –0.42, p = 0.014) and the left pars triangularis (β = –0.44, p = 0.034), indicating an inverse relationship between plasma Aβ40 levels and regional tau deposition (Supplementary Table S1).

**Associations between plasma Aβ42 and tau PET**

In the MCI group, significant negative associations were observed in the corpus callosum and cerebral white matter (β = –0.28, p = 0.002), as well as in multiple cortical regions, including the lateral occipital cortex (β = –0.28, p = 0.001), orbitofrontal cortex (β = –0.20 to –0.26, p = 0.003–0.023), superior frontal cortex (β = –0.25, p = 0.004), superior parietal cortex (β = –0.25, p = 0.002), and superior temporal cortex (β = –0.28, p = 0.002). Additional associations were identified in the precentral and postcentral gyri, lingual gyrus, pars orbitalis, pars triangularis, frontal pole, temporal pole, thalamus, and nucleus accumbens (β range –0.19 to –0.27, p = 0.003–0.040). A single positive association was observed in the fourth ventricle (β = 0.20, p = 0.030).

In the AD group, lower Aβ42 was strongly associated with higher tau deposition in the caudal middle frontal cortex (β = –0.41, p = 0.014), superior frontal cortex (β = –0.47, p = 0.020), parahippocampal cortex (β = –0.42, p = 0.042), posterior cingulate cortex (β = –0.45, p = 0.016), and precuneus (β = –0.46, p = 0.032). Additional significant associations were found in the middle temporal gyrus (β = –0.31 to –0.32, p = 0.041–0.048), banks of the superior temporal sulcus (β = –0.30, p = 0.049), and pars triangularis (β = –0.41, p = 0.048) (Supplementary Table S2).

**Associations between plasma Aβ42/Aβ40 ratio and tau PET**

In CN individuals, lower Aβ42/Aβ40 was associated with greater tau burden in the bilateral amygdala (β = –0.22, p = 0.011), the left amygdala (β = –0.21, p = 0.015), and the right amygdala (β = –0.19, p = 0.030).

In the MCI group, widespread associations were observed, with significant negative correlations across the corpus callosum (β = –0.29, p = 0.002), cerebral white matter (β = –0.29, p = 0.002), and multiple cortical regions, including the lateral occipital cortex (β = –0.33 to –0.34, p < 0.001), lateral orbitofrontal cortex (β = –0.28, p = 0.001), lingual gyrus (β = –0.24 to –0.29, p = 0.001–0.015), superior frontal cortex (β = –0.34 to –0.36, p < 0.001), superior parietal cortex (β = –0.34, p < 0.001), and superior temporal cortex (β = –0.25, p = 0.003). Additional associations were observed in the pars opercularis, pars orbitalis, pars triangularis, precentral and postcentral cortices, insula, cingulate cortex, thalamus, and temporal pole (β range –0.19 to –0.34, p = 0.001–0.040).

In AD participants, significant associations were detected in the bilateral entorhinal cortex (β range –0.37 to –0.38, p = 0.027–0.039), the right lingual gyrus (β = –0.36, p = 0.045), the bilateral rostral middle frontal cortex (β = –0.38, p = 0.038), and the bilateral hippocampus (β range –0.40 to –0.43, p = 0.026–0.030) (Supplementary Table S3).

**Associations between plasma GFAP and tau PET**

In the MCI group, plasma GFAP showed robust positive associations with tau PET uptake across frontal, parietal, and occipital cortices, as well as selected subcortical regions. The strongest associations were observed in the bilateral caudal middle frontal (β = 0.37, p < 0.001), rostral middle frontal (β = 0.33–0.35, p ≤ 0.001), and superior frontal cortices (β = 0.32–0.33, p ≤ 0.001). Additional significant correlations were identified in the lateral orbitofrontal cortex (β = 0.31, p = 0.001), lingual gyrus (β = 0.22–0.26, p = 0.010–0.042), orbitofrontal pars regions (β = 0.19–0.24, p = 0.014–0.043), pars triangularis (β = 0.22, p = 0.025), precentral and postcentral gyri (β = 0.22–0.27, p = 0.011–0.035), rostral anterior cingulate (β = 0.23, p = 0.027), and frontal pole (β = 0.20, p = 0.042). Subcortical associations included the left accumbens area (β = 0.25, p = 0.023).

In contrast, negative associations were observed between GFAP and tau burden in cerebrospinal fluid spaces, including the third (β = –0.19, p = 0.043) and fourth ventricles (β = –0.21, p = 0.031) (Supplementary Table S4).

**Associations between plasma NfL and tau PET**

In the MCI group, higher plasma NfL was positively associated with tau PET uptake in multiple callosal subregions (β = 0.24, p = 0.022) and cerebral white matter (β = 0.24, p = 0.022). Additional significant associations were observed in the bilateral caudal middle frontal cortex (β = 0.23, p = 0.016), left pars opercularis (β = 0.22, p = 0.031), right lingual gyrus (β = 0.29, p = 0.009), bilateral lingual cortex (β = 0.31, p = 0.006), and right caudal middle frontal cortex (β = 0.27, p = 0.006).

In the AD group, stronger associations were observed between NfL and medial temporal as well as subcortical tau burden, including the bilateral accumbens area (β = 0.54, p = 0.014), bilateral amygdala (β = 0.49, p = 0.049), bilateral hippocampus (β = 0.46, p = 0.027), bilateral temporal pole (β = 0.43, p = 0.031), left amygdala (β = 0.56, p = 0.013), left hippocampus (β = 0.43, p = 0.040), left accumbens area (β = 0.49, p = 0.048), and right accumbens area (β = 0.58, p = 0.009) (Supplementary Table S5).

**Associations between plasma p-tau217 and tau PET**

In the MCI group, plasma p-tau217 showed widespread positive associations with tau PET uptake across callosal and cortical regions, including the corpus callosum (β = 0.29, p = 0.002), cerebral white matter (β = 0.29, p = 0.002), and accumbens area (β = 0.23, p = 0.015). Strong cortical associations included the caudal middle frontal cortex (β = 0.54, p < 0.001), pars opercularis (β = 0.56, p < 0.001), pars triangularis (β = 0.46, p < 0.001), superior frontal cortex (β = 0.51, p < 0.001), and temporal pole (β = 0.44, p < 0.001).

In the AD group, p-tau217 demonstrated robust associations with tau PET uptake in medial temporal, limbic, and association cortices, including the middle temporal gyrus (β = 0.71, p < 0.001), supramarginal gyrus (β = 0.71, p < 0.001), temporal pole (β = 0.56, p = 0.001), hippocampus (β = 0.60, p = 0.002), entorhinal cortex (β range 0.50–0.57, p ≤ 0.005), amygdala (β = 0.54, p = 0.008), superior frontal cortex (β = 0.48–0.61, p ≤ 0.014), orbitofrontal cortex (β = 0.52, p = 0.019), and rostral anterior cingulate cortex (β = 0.47–0.56, p ≤ 0.038) (Supplementary Table S6).

**Associations between plasma p-tau217/Aβ40 ratio and tau PET**

In the MCI group, higher plasma p-tau217/Aβ40 ratios were significantly associated with greater tau PET uptake across widespread subcortical, callosal, and cortical regions, including the supramarginal gyrus (β = 0.68, p < 0.001), caudal middle frontal cortex (β = 0.47, p < 0.001), pars opercularis (β = 0.48, p < 0.001), superior frontal cortex (β = 0.45, p < 0.001), and additional orbitofrontal, temporal, and cingulate regions (β = 0.27–0.45, p ≤ 0.025).

In the AD group, strong associations were observed in the banks of the superior temporal sulcus (β = 0.80, p < 0.001), inferior parietal cortex (β = 0.66, p = 0.001), supramarginal gyrus (β = 0.71, p = 0.001), temporal pole (β = 0.54, p = 0.001), entorhinal cortex (β = 0.47–0.48, p ≈ 0.015), hippocampus (β = 0.42–0.44, p ≈ 0.026–0.040), amygdala (β = 0.43–0.45, p ≈ 0.041–0.049), fusiform gyrus (β = 0.47, p = 0.019), insula (β = 0.47, p = 0.011), and frontal regions (β = 0.43–0.69, p ≤ 0.047) (Supplementary Table S7).

**Supplementary Table 1.** Significant associations between Aβ40 and tau PET findings

| Diagnosis | ROIs | Standardized β | t-value | F-value | Adjusted R² | 95% CI | | P-value |
| --- | --- | --- | --- | --- | --- | --- | --- | --- |
|  |  |  |  |  |  | **lower** | **upper** |  |
| AD | ctx-lh-caudalmiddlefrontal | -0.42 | -2.80 | 4.98 | 0.31 | -0.01 | 0.00 | 0.014 |
|  | ctx-lh-parstriangularis | -0.44 | -2.76 | 3.34 | 0.22 | 0.00 | 0.00 | 0.034 |

**Supplementary Table 2.** Significant associations between Aβ42 and tau PET findings

| Diagnosis | ROIs | Standardized β | t-value | F-value | Adjusted R² | 95% CI | | P-Value |
| --- | --- | --- | --- | --- | --- | --- | --- | --- |
|  |  |  |  |  |  | Upper | Lower |  |
| MCI | cc-anterior | -0.28 | -3.41 | 4.23 | 0.08 | -0.01 | 0.00 | 0.002 |
|  | cc-central | -0.28 | -3.41 | 4.23 | 0.08 | -0.01 | 0.00 | 0.002 |
|  | cc-mid-anterior | -0.28 | -3.41 | 4.23 | 0.08 | -0.01 | 0.00 | 0.002 |
|  | cc-mid-posterior | -0.28 | -3.41 | 4.23 | 0.08 | -0.01 | 0.00 | 0.002 |
|  | cc-posterior | -0.28 | -3.41 | 4.23 | 0.08 | -0.01 | 0.00 | 0.002 |
|  | bilateral-cerebral-white-matter | -0.28 | -3.41 | 4.23 | 0.08 | -0.01 | 0.00 | 0.002 |
|  | ctx-bilateral-lateraloccipital | -0.28 | -3.42 | 7.39 | 0.17 | -0.01 | 0.00 | 0.001 |
|  | ctx-lh-lateraloccipital | -0.24 | -2.88 | 6.22 | 0.14 | -0.01 | 0.00 | 0.005 |
|  | ctx-lh-lateralorbitofrontal | -0.20 | -2.39 | 4.17 | 0.09 | -0.01 | 0.00 | 0.023 |
|  | ctx-lh-parsorbitalis | -0.26 | -3.21 | 5.84 | 0.12 | -0.01 | 0.00 | 0.003 |
|  | ctx-lh-parstriangularis | -0.21 | -2.56 | 4.52 | 0.09 | -0.01 | 0.00 | 0.020 |
|  | ctx-lh-postcentral | -0.20 | -2.28 | 2.77 | 0.05 | -0.01 | 0.00 | 0.035 |
|  | ctx-lh-precentral | -0.21 | -2.47 | 3.36 | 0.07 | -0.01 | 0.00 | 0.021 |
|  | ctx-lh-rostralmiddlefrontal | -0.19 | -2.21 | 4.79 | 0.11 | -0.01 | 0.00 | 0.041 |
|  | ctx-lh-superiorfrontal | -0.25 | -2.96 | 5.08 | 0.11 | -0.01 | 0.00 | 0.004 |
|  | ctx-bilateral-lingual | -0.20 | -2.40 | 6.83 | 0.15 | -0.01 | 0.00 | 0.021 |
|  | ctx-bilateral-parsorbitalis | -0.27 | -3.25 | 5.01 | 0.10 | -0.01 | 0.00 | 0.003 |
|  | ctx-bilateral-parstriangularis | -0.25 | -3.00 | 5.33 | 0.11 | -0.01 | 0.00 | 0.006 |
|  | ctx-bilateral-postcentral | -0.25 | -3.05 | 5.08 | 0.11 | -0.01 | 0.00 | 0.005 |
|  | ctx-bilateral-precentral | -0.22 | -2.50 | 3.20 | 0.06 | -0.01 | 0.00 | 0.024 |
|  | ctx-rh-frontalpole | -0.22 | -2.63 | 3.82 | 0.08 | -0.01 | 0.00 | 0.017 |
|  | ctx-rh-lingual | -0.21 | -2.48 | 6.45 | 0.15 | -0.01 | 0.00 | 0.022 |
|  | ctx-rh-paracentral | -0.22 | -2.67 | 3.09 | 0.06 | -0.01 | 0.00 | 0.026 |
|  | ctx-rh-postcentral | -0.20 | -2.29 | 2.93 | 0.06 | -0.01 | 0.00 | 0.040 |
|  | ctx-rh-postcentral | -0.21 | -2.46 | 3.63 | 0.07 | -0.01 | 0.00 | 0.027 |
|  | ctx-rh-superiorfrontal | -0.25 | -2.86 | 4.87 | 0.11 | -0.01 | 0.00 | 0.007 |
|  | ctx-rh-superiorparietal | -0.25 | -3.24 | 9.44 | 0.21 | -0.01 | 0.00 | 0.002 |
|  | ctx-rh-superiortemporal | -0.28 | -3.32 | 7.42 | 0.17 | -0.01 | 0.00 | 0.002 |
|  | ctx-bilateral-temporalpole | -0.19 | -2.19 | 3.94 | 0.08 | -0.01 | 0.00 | 0.038 |
|  | left-cerebral-white-matter | -0.28 | -3.41 | 4.23 | 0.08 | -0.01 | 0.00 | 0.002 |
|  | right-accumbens-area | -0.20 | -2.44 | 4.13 | 0.08 | -0.03 | 0.00 | 0.037 |
|  | right-cerebral-white-matter | -0.28 | -3.41 | 4.23 | 0.08 | -0.01 | 0.00 | 0.002 |
|  | 4th-ventricle | 0.20 | 2.39 | 3.22 | 0.06 | 0.00 | 0.01 | 0.030 |
| AD | ctx-lh-caudalmiddlefrontal | -0.41 | -2.61 | 4.06 | 0.27 | -0.07 | -0.01 | 0.014 |
|  | ctx-lh-middletemporal | -0.32 | -2.13 | 4.42 | 0.28 | -0.09 | 0.00 | 0.041 |
|  | ctx-lh-parstriangularis | -0.41 | -2.33 | 3.04 | 0.20 | -0.03 | 0.00 | 0.048 |
|  | ctx-lh-superiorfrontal | -0.47 | -2.46 | 2.03 | 0.11 | -0.04 | 0.00 | 0.020 |
|  | ctx-bilateral-middletemporal | -0.31 | -2.06 | 4.21 | 0.27 | -0.09 | 0.00 | 0.048 |
|  | ctx-bilateral-parahippocampal | -0.41 | -2.73 | 4.21 | 0.26 | -0.07 | -0.01 | 0.041 |
|  | ctx-bilateral-posteriorcingulate | -0.45 | -2.57 | 3.49 | 0.24 | -0.07 | -0.01 | 0.016 |
|  | ctx-bilateral-precuneus | -0.46 | -2.58 | 2.71 | 0.18 | -0.05 | -0.01 | 0.032 |
|  | ctx-rh-bankssts | -0.30 | -2.04 | 4.96 | 0.31 | -0.15 | 0.00 | 0.049 |
|  | ctx-rh-parahippocampal | -0.42 | -2.71 | 3.58 | 0.22 | -0.08 | -0.01 | 0.042 |

**Supplementary Table 3.** Significant associations between Aβ42/Aβ40 and tau PET findings

| Diagnosis | ROIs | Standardized β | t-value | F-value | Adjusted R² | 95% CI | | P-Value |
| --- | --- | --- | --- | --- | --- | --- | --- | --- |
|  |  |  |  |  |  | **lower** | **upper** |  |
| CN | bilateral-amygdala | -0.22 | -3.17 | 5.15 | 0.08 | -4.42 | -1.03 | 0.011 |
|  | left-amygdala | -0.21 | -3.06 | 4.20 | 0.06 | -4.80 | -1.03 | 0.015 |
|  | right-amygdala | -0.19 | -2.84 | 4.98 | 0.07 | -4.18 | -0.76 | 0.030 |
| MCI | cc-anterior | -0.29 | -3.63 | 4.63 | 0.09 | -5.59 | -1.65 | 0.002 |
|  | cc-central | -0.29 | -3.63 | 4.63 | 0.09 | -5.59 | -1.65 | 0.002 |
|  | cc-mid-anterior | -0.29 | -3.63 | 4.63 | 0.09 | -5.59 | -1.65 | 0.002 |
|  | cc-mid-posterior | -0.29 | -3.63 | 4.63 | 0.09 | -5.59 | -1.65 | 0.002 |
|  | cc-posterior | -0.29 | -3.63 | 4.63 | 0.09 | -5.59 | -1.65 | 0.002 |
|  | cerebral white matter | -0.29 | -3.63 | 4.63 | 0.09 | -5.59 | -1.65 | 0.002 |
|  | ctx-bilateral-lateraloccipital | -0.33 | -4.24 | 9.14 | 0.21 | -5.51 | -2.00 | <0.001 |
|  | ctx-lh-lateraloccipital | -0.34 | -4.50 | 9.90 | 0.22 | -6.11 | -2.38 | <0.001 |
|  | ctx-lh-lateralorbitofrontal | -0.28 | -3.52 | 6.05 | 0.13 | -6.05 | -1.70 | 0.001 |
|  | ctx-lh-lingual | -0.24 | -3.13 | 7.56 | 0.17 | -3.95 | -0.89 | 0.015 |
|  | ctx-lh-parsopercularis | -0.20 | -2.43 | 6.43 | 0.15 | -3.47 | -0.36 | 0.029 |
|  | ctx-lh-parsorbitalis | -0.28 | -3.53 | 6.55 | 0.14 | -6.17 | -1.73 | 0.001 |
|  | ctx-lh-parstriangularis | -0.25 | -3.11 | 5.49 | 0.12 | -4.80 | -1.07 | 0.005 |
|  | ctx-lh-postcentral | -0.26 | -3.10 | 4.09 | 0.09 | -3.17 | -0.70 | 0.008 |
|  | ctx-lh-precentral | -0.27 | -3.33 | 4.85 | 0.10 | -3.66 | -0.93 | 0.003 |
|  | ctx-lh-rostralanteriorcingulate | -0.19 | -2.36 | 2.16 | 0.03 | -3.86 | -0.34 | 0.039 |
|  | ctx-lh-rostralmiddlefrontal | -0.33 | -4.07 | 8.29 | 0.18 | -5.49 | -1.90 | <0.001 |
|  | ctx-lh-superiorfrontal | -0.36 | -4.61 | 8.71 | 0.19 | -6.01 | -2.40 | <0.001 |
|  | ctx-bilateral-lingual | -0.29 | -3.83 | 9.70 | 0.21 | -4.45 | -1.42 | 0.001 |
|  | ctx-bilateral-parsorbitalis | -0.30 | -3.73 | 5.91 | 0.12 | -6.08 | -1.87 | 0.001 |
|  | ctx-bilateral-parstriangularis | -0.24 | -3.02 | 5.54 | 0.12 | -4.55 | -0.95 | 0.006 |
|  | ctx-bilateral-postcentral | -0.29 | -3.70 | 6.50 | 0.14 | -3.67 | -1.11 | 0.001 |
|  | ctx-bilateral-precentral | -0.27 | -3.27 | 4.54 | 0.10 | -3.54 | -0.87 | 0.003 |
|  | ctx-rh-frontalpole | -0.25 | -3.08 | 4.76 | 0.10 | -6.04 | -1.32 | 0.006 |
|  | ctx-rh-insula | -0.23 | -2.86 | 4.41 | 0.09 | -3.54 | -0.64 | 0.012 |
|  | ctx-rh-lingual | -0.27 | -3.47 | 8.37 | 0.19 | -4.23 | -1.16 | 0.002 |
|  | ctx-rh-paracentral | -0.26 | -3.16 | 3.93 | 0.08 | -3.79 | -0.87 | 0.012 |
|  | ctx-rh-postcentral | -0.21 | -2.56 | 3.51 | 0.07 | -2.65 | -0.34 | 0.040 |
|  | ctx-rh-perecentral | -0.22 | -2.67 | 4.12 | 0.09 | -3.16 | -0.47 | 0.021 |
|  | ctx-rh-superiorfrontal | -0.34 | -4.28 | 7.88 | 0.18 | -5.07 | -1.86 | <0.001 |
|  | ctx-rh-superiorparietal | -0.34 | -4.59 | 12.83 | 0.27 | -5.73 | -2.28 | <0.001 |
|  | ctx-rh-superiortemporal | -0.25 | -3.11 | 7.19 | 0.16 | -3.85 | -0.86 | 0.003 |
|  | ctx-rh-temporalpole | -0.22 | -2.69 | 4.39 | 0.09 | -5.93 | -0.90 | 0.011 |
|  | ctx-bilateral-superiorfrontal | -0.35 | -4.49 | 8.43 | 0.18 | -5.72 | -2.22 | <0.001 |
|  | ctx-bilateral-temporalpole | -0.25 | -3.14 | 5.41 | 0.12 | -6.21 | -1.41 | 0.003 |
|  | left-cerebral-white-matter | -0.29 | -3.63 | 4.63 | 0.09 | -5.59 | -1.65 | 0.002 |
|  | right-cerebral-white-matter | -0.29 | -3.63 | 4.63 | 0.09 | -5.59 | -1.65 | 0.002 |
|  | right-thalamus-proper | -0.25 | -3.08 | 5.19 | 0.10 | -4.44 | -0.96 | 0.018 |
|  | left-thalamus-proper | -0.24 | -3.06 | 5.12 | 0.10 | -4.37 | -0.94 | 0.011 |
| AD | ctx-entorhinal | -0.38 | -2.57 | 4.10 | 0.25 | -56.32 | -6.60 | 0.034 |
|  | ctx-rh-entorhinal | -0.37 | -2.52 | 3.79 | 0.23 | -61.14 | -6.55 | 0.039 |
|  | ctx-rh-lingual | -0.36 | -2.31 | 3.35 | 0.22 | -28.20 | -1.71 | 0.045 |
|  | ctx-bilateral-rostralmiddlefrontal | -0.38 | -2.49 | 3.65 | 0.25 | -13.94 | -1.36 | 0.038 |
|  | ctx-lh-entorhinal | -0.37 | -2.60 | 4.61 | 0.28 | -56.05 | -6.87 | 0.027 |
|  | bilateral-hippocampus | -0.43 | -2.79 | 2.86 | 0.16 | -32.86 | -5.18 | 0.027 |
|  | left-hippocampus | -0.43 | -2.79 | 2.89 | 0.17 | -34.04 | -5.33 | 0.030 |
|  | right-hippocampus | -0.40 | -2.59 | 2.55 | 0.14 | -32.51 | -3.90 | 0.026 |

**Supplementary Table 4.** Significant associations between GFAP and tau PET findings

| Diagnosis | ROIs | Standardized β | t-value | F-value | Adjusted R² | 95% CI | | P-Value |
| --- | --- | --- | --- | --- | --- | --- | --- | --- |
|  |  |  |  |  |  | **Upper** | **Lower** |  |
| MCI | csf | -0.20 | -2.17 | 3.28 | 0.06 | 0.00 | 0.00 | 0.032 |
|  | ctx-bilateral-caudalmiddlefrontal | 0.37 | 4.22 | 8.97 | 0.21 | 0.00 | 0.00 | <0.001 |
|  | ctx-lh-caudalanteriorcingulate | 0.22 | 2.28 | 1.81 | 0.02 | 0.00 | 0.00 | 0.049 |
|  | ctx-lh-parsopercularis | 0.24 | 2.53 | 4.83 | 0.11 | 0.00 | 0.00 | 0.029 |
|  | ctx-lh-parsorbitalis | 0.19 | 2.18 | 4.03 | 0.08 | 0.00 | 0.00 | 0.043 |
|  | ctx-lh-postcentral | 0.23 | 2.27 | 2.30 | 0.04 | 0.00 | 0.00 | 0.035 |
|  | ctx-lh-precentral | 0.27 | 2.79 | 3.09 | 0.06 | 0.00 | 0.00 | 0.011 |
|  | ctx-lh-rostralmiddlefrontal | 0.33 | 3.59 | 5.89 | 0.14 | 0.00 | 0.00 | 0.001 |
|  | ctx-lh-soperiorfrontal | 0.32 | 3.38 | 4.81 | 0.11 | 0.00 | 0.00 | 0.001 |
|  | ctx-bilateral-lingual | 0.26 | 2.80 | 5.65 | 0.13 | 0.00 | 0.00 | 0.010 |
|  | ctx-bilateral-parsorbitalis | 0.24 | 2.59 | 3.52 | 0.07 | 0.00 | 0.00 | 0.014 |
|  | ctx-bilateral-partstriangularis | 0.22 | 2.40 | 3.39 | 0.07 | 0.00 | 0.00 | 0.025 |
|  | ctx-bilateral-precentral | 0.22 | 2.27 | 2.41 | 0.04 | 0.00 | 0.00 | 0.035 |
|  | ctx-rh-frontalpole | 0.20 | 2.19 | 3.84 | 0.08 | 0.00 | 0.00 | 0.042 |
|  | ctx-rh-lateralorbitofrontal | 0.31 | 3.36 | 4.87 | 0.11 | 0.00 | 0.00 | 0.001 |
|  | ctx-rh-lingual | 0.22 | 2.14 | 4.02 | 0.09 | 0.00 | 0.00 | 0.042 |
|  | ctx-rh-parsorbitalis | 0.21 | 2.29 | 2.35 | 0.04 | 0.00 | 0.00 | 0.036 |
|  | ctx-rh-rostralmiddlefrontal | 0.35 | 3.93 | 5.58 | 0.13 | 0.00 | 0.00 | <0.001 |
|  | ctx-bilateral-rostralanteriorcingulate | 0.23 | 2.23 | 1.59 | 0.02 | 0.00 | 0.00 | 0.027 |
|  | ctx-bilateral-rostralmiddlefrontal | 0.35 | 4.08 | 7.21 | 0.17 | 0.00 | 0.00 | <0.001 |
|  | ctx-bilateral-superiorfrontal | 0.33 | 3.62 | 5.39 | 0.12 | 0.00 | 0.00 | <0.001 |
|  | left-accumbens-area | 0.25 | 2.77 | 4.21 | 0.09 | 0.00 | 0.00 | 0.023 |
|  | 3rd-ventricle | -0.19 | -2.04 | 3.51 | 0.07 | 0.00 | 0.00 | 0.043 |
|  | 4th-ventricle | -0.21 | -2.27 | 3.17 | 0.06 | 0.00 | 0.00 | 0.031 |

**Supplementary Table 5.** Significant associations between NfL and tau PET findings

| Diagnosis | ROIs | Standardized β | t-value | F-value | Adjusted R² | 95% CI | | P-Value |
| --- | --- | --- | --- | --- | --- | --- | --- | --- |
|  |  |  |  |  |  | **Upper** | **Lower** |  |
| MCI | cc-anterior | 0.24 | 2.45 | 3.32 | 0.06 | 0.00 | 0.01 | 0.022 |
|  | cc-central | 0.24 | 2.45 | 3.32 | 0.06 | 0.00 | 0.01 | 0.022 |
|  | cc-mid-anterior | 0.24 | 2.45 | 3.32 | 0.06 | 0.00 | 0.01 | 0.022 |
|  | cc-mid-posterior | 0.24 | 2.45 | 3.32 | 0.06 | 0.00 | 0.01 | 0.022 |
|  | cc-anterior | 0.24 | 2.45 | 3.32 | 0.06 | 0.00 | 0.01 | 0.022 |
|  | bilateral-cerebral-white-matter | 0.24 | 2.45 | 3.32 | 0.06 | 0.00 | 0.01 | 0.022 |
|  | ctx-bilateral-caudalmiddlefrontal | 0.23 | 2.44 | 6.03 | 0.14 | 0.00 | 0.01 | 0.016 |
|  | ctx-lh-parsopercularis | 0.22 | 2.32 | 4.93 | 0.11 | 0.00 | 0.01 | 0.031 |
|  | ctx-rh-lingual | 0.29 | 2.88 | 6.31 | 0.15 | 0.00 | 0.01 | 0.009 |
|  | left-cerebral-white-matter | 0.24 | 2.45 | 3.32 | 0.06 | 0.00 | 0.01 | 0.022 |
|  | right-cerebral-white-matter | 0.24 | 2.45 | 3.32 | 0.06 | 0.00 | 0.01 | 0.022 |
|  | ctx-bilateral-lingual | 0.31 | 3.09 | 7.15 | 0.17 | 0.00 | 0.01 | 0.006 |
|  | ctx-rh-caudalmiddlefrontal | 0.27 | 2.80 | 6.25 | 0.15 | 0.00 | 0.01 | 0.006 |
| AD | bilateral-accumbens-area | 0.54 | 3.36 | 4.67 | 0.28 | 0.01 | 0.05 | 0.014 |
|  | bilateral-amygdala | 0.49 | 2.74 | 2.92 | 0.17 | 0.01 | 0.07 | 0.049 |
|  | ctx-bilateral-temporalpole | 0.43 | 2.61 | 5.31 | 0.32 | 0.01 | 0.04 | 0.031 |
|  | bilateral-hippocampus | 0.46 | 2.55 | 2.24 | 0.12 | 0.00 | 0.04 | 0.027 |
|  | left-accumbens-area | 0.49 | 2.88 | 5.19 | 0.31 | 0.01 | 0.06 | 0.048 |
|  | left-amygdala | 0.56 | 3.24 | 3.78 | 0.23 | 0.02 | 0.08 | 0.013 |
|  | left-hippocampus | 0.43 | 2.38 | 2.10 | 0.10 | 0.00 | 0.04 | 0.040 |
|  | right-accumbens-area | 0.58 | 3.52 | 3.86 | 0.24 | 0.01 | 0.05 | 0.009 |
|  | right-hippocampus | 0.47 | 2.57 | 2.21 | 0.11 | 0.00 | 0.04 | 0.026 |

**Supplementary Table 6.** Significant associations between tau217 and tau PET findings

| Diagnosis | ROIs | Standardized β | t-value | F-value | Adjusted R² | 95% CI | | P-Value |
| --- | --- | --- | --- | --- | --- | --- | --- | --- |
|  |  |  |  |  |  | **Upper** | **Lower** |  |
| MCI | bilateral-accumbens-area | 0.23 | 2.98 | 5.70 | 0.12 | 0.13 | 0.64 | 0.015 |
|  | cc-anterior | 0.29 | 3.49 | 4.79 | 0.10 | 0.08 | 0.29 | 0.002 |
|  | cc-central | 0.29 | 3.49 | 4.79 | 0.10 | 0.08 | 0.29 | 0.002 |
|  | cc-mid-anterior | 0.29 | 3.49 | 4.79 | 0.10 | 0.08 | 0.29 | 0.002 |
|  | cc-mid-posterior | 0.29 | 3.49 | 4.79 | 0.10 | 0.08 | 0.29 | 0.002 |
|  | cc-posterior | 0.29 | 3.49 | 4.79 | 0.10 | 0.08 | 0.29 | 0.002 |
|  | bilateral-cerebral-white-matter | 0.29 | 3.49 | 4.79 | 0.10 | 0.08 | 0.29 | 0.002 |
|  | ctx-bilateral-caudalmiddlefrontal | 0.54 | 5.63 | 16.34 | 0.33 | 0.22 | 0.45 | <0.001 |
|  | ctx-bilateral-frontalpole | 0.33 | 3.98 | 6.20 | 0.14 | 0.11 | 0.34 | 0.001 |
|  | ctx-bilateral-lateraloccipital | 0.42 | 4.25 | 8.83 | 0.20 | 0.13 | 0.35 | <0.001 |
|  | ctx-bilateral-lateralorbitofrontal | 0.42 | 4.66 | 8.61 | 0.19 | 0.17 | 0.41 | <0.001 |
|  | ctx-lh-caudalanteriorcingulate | 0.29 | 3.17 | 3.61 | 0.07 | 0.06 | 0.25 | 0.012 |
|  | ctx-lh-lateraloccipital | 0.40 | 3.95 | 8.42 | 0.19 | 0.13 | 0.38 | <0.001 |
|  | ctx-lh-lateralorbitofrontal | 0.34 | 3.83 | 6.94 | 0.15 | 0.12 | 0.37 | 0.001 |
|  | ctx-lh-medialorbitofrontal | 0.34 | 3.85 | 7.00 | 0.15 | 0.11 | 0.34 | <0.001 |
|  | ctx-lh-parsopercularis | 0.45 | 4.36 | 10.79 | 0.24 | 0.12 | 0.32 | <0.001 |
|  | ctx-lh-parsorbitalis | 0.43 | 5.29 | 10.42 | 0.22 | 0.19 | 0.43 | <0.001 |
|  | ctx-lh-parstriangularis | 0.43 | 4.94 | 9.53 | 0.20 | 0.16 | 0.37 | <0.001 |
|  | ctx-lh-postcentral | 0.35 | 4.07 | 5.87 | 0.13 | 0.07 | 0.20 | 0.001 |
|  | ctx-lh-precentral | 0.44 | 5.36 | 10.23 | 0.22 | 0.12 | 0.26 | <0.001 |
|  | ctx-lh-rostralmiddlefrontal | 0.43 | 4.28 | 9.38 | 0.21 | 0.13 | 0.36 | <0.001 |
|  | ctx-lh-superiorfrontal | 0.51 | 5.94 | 13.50 | 0.28 | 0.20 | 0.40 | <0.001 |
|  | ctx-lh-temporalpole | 0.43 | 5.34 | 9.00 | 0.20 | 0.25 | 0.55 | <0.001 |
|  | ctx-bilateral-lingual | 0.26 | 2.75 | 7.50 | 0.17 | 0.04 | 0.23 | 0.010 |
|  | ctx-bilateral-parsopercularis | 0.56 | 6.46 | 15.79 | 0.31 | 0.22 | 0.41 | <0.001 |
|  | ctx-bilateral-parstraingularis | 0.46 | 5.25 | 10.74 | 0.23 | 0.17 | 0.37 | <0.001 |
|  | ctx-bilateral-postcentral | 0.32 | 3.76 | 6.94 | 0.15 | 0.06 | 0.20 | 0.001 |
|  | ctx-bilateral-precentral | 0.35 | 3.93 | 6.62 | 0.15 | 0.07 | 0.22 | <0.001 |
|  | ctx-rh-frontalpole | 0.35 | 4.31 | 7.54 | 0.16 | 0.14 | 0.39 | <0.001 |
|  | ctx-rh-insula | 0.34 | 3.52 | 6.24 | 0.14 | 0.07 | 0.24 | 0.004 |
|  | ctx-rh-lateralorbitofrontal | 0.48 | 5.69 | 11.40 | 0.24 | 0.23 | 0.48 | <0.001 |
|  | ctx-rh-medialorbitofrontal | 0.37 | 4.18 | 6.93 | 0.15 | 0.12 | 0.33 | <0.001 |
|  | ctx-rh-parsopercularis | 0.34 | 3.33 | 8.48 | 0.19 | 0.08 | 0.31 | 0.001 |
|  | ctx-rh-traingularis | 0.42 | 4.69 | 8.62 | 0.19 | 0.14 | 0.34 | <0.001 |
|  | ctx-rh-precentral | 0.26 | 2.76 | 4.64 | 0.10 | 0.03 | 0.19 | 0.021 |
|  | ctx-rh-rostralmiddlefrontal | 0.40 | 3.75 | 6.26 | 0.15 | 0.11 | 0.37 | <0.001 |
|  | ctx-rh-superiortemporal | 0.42 | 4.05 | 9.33 | 0.21 | 0.10 | 0.30 | <0.001 |
|  | ctx-rh-temporalpole | 0.44 | 5.14 | 9.37 | 0.20 | 0.21 | 0.48 | <0.001 |
|  | ctx-bilateral-rostralanteriorcingulate | 0.26 | 2.91 | 2.71 | 0.05 | 0.04 | 0.20 | 0.009 |
|  | ctx-bilateral-superiorfrontal | 0.48 | 5.16 | 10.29 | 0.23 | 0.17 | 0.38 | <0.001 |
|  | ctx-bilateral-temporalpole | 0.44 | 5.25 | 10.01 | 0.22 | 0.21 | 0.47 | <0.001 |
|  | left-accumbens-area | 0.23 | 2.82 | 4.43 | 0.09 | 0.13 | 0.72 | 0.023 |
|  | left-cerebral-white-matter | 0.29 | 3.49 | 4.79 | 0.10 | 0.08 | 0.29 | 0.002 |
|  | right-accumbens-area | 0.24 | 3.12 | 5.97 | 0.13 | 0.14 | 0.62 | 0.016 |
|  | right-cerebral-white-matter | 0.29 | 3.49 | 4.79 | 0.10 | 0.08 | 0.29 | 0.002 |
|  | 3rd-ventricle | -0.23 | -2.74 | 4.23 | 0.09 | -0.17 | -0.03 | 0.010 |
|  | 4th-ventricle | -0.23 | -2.69 | 3.54 | 0.07 | -0.16 | -0.02 | 0.020 |
| AD | ctx-bilateral-bankssts | 0.49 | 2.91 | 3.17 | 0.21 | 0.24 | 1.39 | 0.024 |
|  | ctx-bilateral-entorhinal | 0.56 | 4.13 | 7.57 | 0.40 | 0.56 | 1.66 | 0.002 |
|  | ctx-lh-entorhinal | 0.50 | 3.65 | 7.09 | 0.38 | 0.44 | 1.56 | 0.005 |
|  | ctx-lh-fusiform | 0.45 | 3.12 | 4.06 | 0.25 | 0.38 | 1.79 | 0.019 |
|  | ctx-lh-parstriangularis | 0.42 | 2.55 | 2.98 | 0.18 | 0.05 | 0.46 | 0.037 |
|  | ctx-lh-superiorfrontal | 0.48 | 2.77 | 2.46 | 0.14 | 0.10 | 0.66 | 0.014 |
|  | ctx-bilateral-parsorbitalis | 0.54 | 3.39 | 4.07 | 0.27 | 0.13 | 0.53 | 0.014 |
|  | ctx-bilateral-precentral | 0.46 | 2.79 | 2.64 | 0.15 | 0.06 | 0.36 | 0.031 |
|  | ctx-rh-entorhinal | 0.57 | 4.19 | 7.46 | 0.40 | 0.63 | 1.82 | 0.001 |
|  | ctx-rh-lateralorbitofrontal | 0.52 | 3.25 | 3.69 | 0.23 | 0.13 | 0.56 | 0.019 |
|  | ctx-rh-middletemporal | 0.71 | 5.77 | 10.39 | 0.51 | 0.98 | 2.05 | <0.001 |
|  | ctx-rh-rostralanteriorcingulate | 0.56 | 3.97 | 6.74 | 0.40 | 0.12 | 0.38 | 0.002 |
|  | ctx-rh-rostralmiddlefrontal | 0.46 | 2.86 | 4.67 | 0.31 | 0.06 | 0.37 | 0.031 |
|  | ctx-bilateral-rostralmiddlefrontal | 0.45 | 2.68 | 4.09 | 0.27 | 0.05 | 0.38 | 0.036 |
|  | ctx-bilateral-superiorfrontal | 0.61 | 3.62 | 3.96 | 0.26 | 0.14 | 0.50 | 0.008 |
|  | ctx-bilateral-superiorparietal | 0.46 | 2.56 | 3.29 | 0.21 | 0.11 | 0.98 | 0.016 |
|  | ctx-bilateral-supramarginal | 0.71 | 5.57 | 11.15 | 0.54 | 0.40 | 0.86 | <0.001 |
|  | ctx-bilateral-temporalpole | 0.56 | 4.45 | 10.09 | 0.49 | 0.41 | 1.10 | 0.001 |
|  | bilateral-hippocampus | 0.60 | 4.08 | 5.16 | 0.30 | 0.32 | 0.94 | 0.002 |
|  | left-hippocampus | 0.57 | 3.81 | 4.74 | 0.28 | 0.29 | 0.95 | 0.004 |
|  | right-amygdala | 0.54 | 3.55 | 4.51 | 0.26 | 0.42 | 1.55 | 0.008 |
|  | right-hippocampus | 0.60 | 4.09 | 5.05 | 0.29 | 0.32 | 0.96 | 0.002 |
|  | ctx-lh-rostralanteriorcingulate | 0.47 | 2.84 | 2.74 | 0.16 | 0.09 | 0.56 | 0.038 |

**Supplementary Table 7.** Significant associations between tau217/Aβ40 and tau PET findings

| Diagnosis | ROIs | Standardized β | t-value | F-value | Adjusted R² | 95% CI | | P-Value |
| --- | --- | --- | --- | --- | --- | --- | --- | --- |
|  |  |  |  |  |  | **Upper** | **Lower** |  |
| MCI | bilateral-accumbens-area | 0.23 | 2.90 | 6.21 | 0.14 | 3.10 | 16.32 | 0.015 |
|  | cc-anterior | 0.25 | 3.07 | 3.76 | 0.08 | 1.42 | 6.57 | 0.005 |
|  | cc-central | 0.25 | 3.07 | 3.76 | 0.08 | 1.42 | 6.57 | 0.005 |
|  | cc-mid-anterior | 0.25 | 3.07 | 3.76 | 0.08 | 1.42 | 6.57 | 0.005 |
|  | cc-mid-posterior | 0.25 | 3.07 | 3.76 | 0.08 | 1.42 | 6.57 | 0.005 |
|  | cc-posterior | 0.25 | 3.07 | 3.76 | 0.08 | 1.42 | 6.57 | 0.005 |
|  | bilateral-cerebral-white-matter | 0.25 | 3.07 | 3.76 | 0.08 | 1.42 | 6.57 | 0.005 |
|  | csf | -0.27 | -3.34 | 5.42 | 0.12 | -4.61 | -1.18 | 0.002 |
|  | ctx-bilateral-caudalmiddlefrontal | 0.47 | 5.08 | 14.55 | 0.30 | 4.41 | 10.05 | <0.001 |
|  | ctx-bilateral-frontalpole | 0.28 | 3.33 | 3.97 | 0.08 | 1.91 | 7.53 | 0.004 |
|  | ctx-bilateral-lateraloccipital | 0.40 | 4.13 | 8.12 | 0.19 | 2.99 | 8.48 | <0.001 |
|  | ctx-bilateral-latralorbitofrontal | 0.40 | 4.96 | 9.12 | 0.20 | 4.19 | 9.76 | <0.001 |
|  | ctx-lh-caudalanteriorcingulate | 0.23 | 2.64 | 3.03 | 0.06 | 0.77 | 5.32 | 0.028 |
|  | ctx-lh-frontalpole | 0.28 | 3.19 | 3.45 | 0.07 | 1.80 | 7.65 | 0.012 |
|  | ctx-lh-lateraloccipital | 0.40 | 4.39 | 9.48 | 0.21 | 3.47 | 9.16 | <0.001 |
|  | ctx-lh-lateralorbitofrontal | 0.34 | 4.19 | 7.53 | 0.17 | 3.14 | 8.75 | <0.001 |
|  | ctx-lh-lingual | 0.23 | 2.58 | 6.77 | 0.15 | 0.67 | 5.09 | 0.039 |
|  | ctx-lh-medialorbitofrontal | 0.33 | 4.11 | 7.59 | 0.17 | 2.86 | 8.18 | <0.001 |
|  | ctx-lh-parsopercularis | 0.40 | 4.68 | 11.09 | 0.24 | 2.89 | 7.12 | <0.001 |
|  | ctx-lh-parsorbitalis | 0.32 | 4.08 | 7.85 | 0.17 | 2.98 | 8.57 | <0.001 |
|  | ctx-lh-parstraingularis | 0.30 | 3.73 | 7.26 | 0.16 | 2.14 | 6.98 | 0.001 |
|  | ctx-lh-postcentral | 0.21 | 2.59 | 3.26 | 0.07 | 0.47 | 3.55 | 0.025 |
|  | ctx-lh-precentral | 0.34 | 4.24 | 7.32 | 0.17 | 1.98 | 5.44 | <0.001 |
|  | ctx-lh-rostralmiddleforntal | 0.41 | 4.63 | 9.76 | 0.22 | 3.36 | 8.37 | <0.001 |
|  | ctx-lh-superiorfrontal | 0.45 | 5.74 | 12.77 | 0.27 | 4.39 | 9.01 | <0.001 |
|  | ctx-lh-supramarginal | 0.68 | 9.64 | 26.64 | 0.44 | 9.27 | 14.06 | <0.001 |
|  | ctx-lh-temporalpole | 0.35 | 4.30 | 7.42 | 0.17 | 4.41 | 11.94 | <0.001 |
|  | ctx-bilateral-lingual | 0.33 | 3.75 | 9.83 | 0.22 | 2.01 | 6.49 | 0.001 |
|  | ctx-bilateral-medialorbitofrontal | 0.32 | 3.91 | 6.97 | 0.16 | 2.50 | 7.62 | <0.001 |
|  | ctx-bilateral-parsopercularis | 0.48 | 6.27 | 14.61 | 0.30 | 4.62 | 8.89 | <0.001 |
|  | ctx-bilateral-parstriangularis | 0.34 | 4.20 | 8.36 | 0.18 | 2.64 | 7.36 | <0.001 |
|  | ctx-bilateral-precentral | 0.34 | 4.07 | 6.32 | 0.14 | 1.86 | 5.39 | <0.001 |
|  | ctx-rh-frontalpole | 0.27 | 3.27 | 4.73 | 0.10 | 2.00 | 8.12 | 0.005 |
|  | ctx-rh-insula | 0.29 | 3.19 | 5.44 | 0.12 | 1.26 | 5.37 | 0.006 |
|  | ctx-rh-lateralorbitofrontal | 0.38 | 4.80 | 8.74 | 0.19 | 4.24 | 10.19 | <0.001 |
|  | ctx-rh-lingual | 0.35 | 3.96 | 10.31 | 0.24 | 2.24 | 6.72 | 0.001 |
|  | ctx-rh-medialorbitofrontal | 0.31 | 3.74 | 6.12 | 0.14 | 2.31 | 7.51 | <0.001 |
|  | ctx-rh-parsopercularis | 0.36 | 4.24 | 9.95 | 0.22 | 2.78 | 7.65 | <0.001 |
|  | ctx-rh-parstraingularis | 0.23 | 2.68 | 4.34 | 0.09 | 0.95 | 6.29 | 0.025 |
|  | ctx-rh-parsopercularis | 0.32 | 3.96 | 6.76 | 0.15 | 2.37 | 7.10 | <0.001 |
|  | ctx-lh-postcentral | 0.21 | 2.35 | 3.42 | 0.07 | 0.29 | 3.45 | 0.040 |
|  | ctx-lh-precentral | 0.24 | 2.65 | 4.51 | 0.10 | 0.64 | 4.41 | 0.021 |
|  | ctx-lh-rostralmiddlefrontal | 0.40 | 4.42 | 7.41 | 0.17 | 3.36 | 8.80 | <0.001 |
|  | ctx-lh-superiorfrontal | 0.41 | 4.71 | 9.34 | 0.21 | 3.12 | 7.65 | <0.001 |
|  | ctx-lh-superiortemporal | 0.44 | 4.88 | 11.42 | 0.25 | 3.18 | 7.53 | <0.001 |
|  | ctx-lh-temporalpole | 0.37 | 4.44 | 7.98 | 0.18 | 4.16 | 10.85 | <0.001 |
|  | ctx-bilateral-rostralmiddlefrontal | 0.45 | 5.58 | 11.91 | 0.26 | 4.61 | 9.67 | <0.001 |
|  | ctx-bilateral-superiorfrontal | 0.43 | 4.98 | 9.82 | 0.22 | 3.69 | 8.54 | <0.001 |
|  | ctx-bilateral-temporalpole | 0.39 | 4.65 | 8.89 | 0.20 | 4.31 | 10.71 | <0.001 |
|  | left-accumbens-area | 0.20 | 2.49 | 4.30 | 0.09 | 1.99 | 17.32 | 0.033 |
|  | left-cerbral-white-matter | 0.25 | 3.07 | 3.76 | 0.08 | 1.42 | 6.57 | 0.005 |
|  | right-accumbens-area | 0.22 | 2.75 | 5.96 | 0.13 | 2.47 | 15.18 | 0.024 |
|  | right-cerbral-white-matter | 0.25 | 3.07 | 3.76 | 0.08 | 1.42 | 6.57 | 0.005 |
|  | 3rd-ventricle | -0.26 | -3.23 | 5.61 | 0.12 | -4.55 | -1.09 | 0.005 |
|  | 4th-ventricle | -0.26 | -3.21 | 4.81 | 0.11 | -4.43 | -1.05 | 0.008 |
| AD | bilateral-amygdala | 0.43 | 2.52 | 2.80 | 0.15 | 2.98 | 27.59 | 0.049 |
|  | ctx-bilateral-bankssts | 0.80 | 5.34 | 8.74 | 0.48 | 15.87 | 35.59 | <0.001 |
|  | ctx-bilateral-entorhinal | 0.47 | 3.06 | 5.25 | 0.30 | 6.05 | 29.85 | 0.015 |
|  | ctx-bilateral-inferiorparietal | 0.66 | 4.20 | 7.96 | 0.45 | 10.57 | 30.60 | 0.001 |
|  | ctx-lh-entorhinal | 0.43 | 2.78 | 5.24 | 0.30 | 4.47 | 28.52 | 0.026 |
|  | ctx-lh-fusiform | 0.47 | 2.84 | 4.35 | 0.26 | 6.11 | 37.02 | 0.019 |
|  | ctx-lh-inferiorparietal | 0.66 | 4.60 | 9.53 | 0.49 | 15.49 | 40.07 | <0.001 |
|  | ctx-lh-inferiortemporal | 0.49 | 3.51 | 8.45 | 0.43 | 11.72 | 43.82 | 0.007 |
|  | ctx-lh-insula | 0.47 | 3.13 | 5.67 | 0.32 | 3.35 | 15.72 | 0.011 |
|  | ctx-lh-parstraingularis | 0.65 | 3.99 | 5.66 | 0.35 | 3.74 | 11.58 | 0.003 |
|  | ctx-lh-rostralmiddleforntal | 0.69 | 3.99 | 5.14 | 0.33 | 3.83 | 11.87 | 0.002 |
|  | ctx-lh-superiorfrontal | 0.59 | 3.13 | 3.03 | 0.19 | 3.19 | 15.09 | 0.011 |
|  | ctx-lh-superiortemporal | 0.46 | 3.09 | 6.56 | 0.37 | 3.64 | 17.57 | 0.024 |
|  | ctx-bilateral-parsopercularis | 0.72 | 3.92 | 5.03 | 0.33 | 3.86 | 12.26 | 0.003 |
|  | ctx-bilateral-parsorbitalis | 0.53 | 2.81 | 3.06 | 0.20 | 1.71 | 10.85 | 0.030 |
|  | ctx-bilateral-precentral | 0.57 | 3.19 | 3.27 | 0.20 | 1.79 | 8.10 | 0.022 |
|  | ctx-rh-tentorhinal | 0.48 | 3.08 | 5.08 | 0.29 | 6.70 | 32.63 | 0.014 |
|  | ctx-rh-lingual | 0.40 | 2.28 | 3.36 | 0.21 | 0.79 | 14.31 | 0.045 |
|  | ctx-rh-medialorbitofrontal | 0.43 | 2.29 | 1.70 | 0.07 | 0.58 | 10.06 | 0.029 |
|  | ctx-rh-parsorbitalis | 0.60 | 3.04 | 3.41 | 0.23 | 2.18 | 11.13 | 0.030 |
|  | ctx-lh-rostralmiddlefrontal | 0.46 | 2.39 | 3.88 | 0.26 | 0.60 | 7.64 | 0.047 |
|  | ctx-lh-superiortemporal | 0.71 | 4.43 | 5.03 | 0.32 | 5.87 | 15.93 | 0.001 |
|  | ctx-lh-temporalpole | 0.59 | 4.00 | 6.37 | 0.36 | 6.62 | 20.30 | 0.002 |
|  | ctx-bilateral-rostralmiddlefrontal | 0.57 | 3.08 | 4.81 | 0.32 | 1.77 | 8.74 | 0.027 |
|  | ctx-bilateral-superiorfrontal | 0.66 | 3.36 | 3.49 | 0.23 | 2.64 | 10.85 | 0.008 |
|  | ctx-bilateral-temporalpole | 0.54 | 3.97 | 8.79 | 0.44 | 6.90 | 21.34 | 0.001 |
|  | bilateral-hippocampus | 0.44 | 2.56 | 2.42 | 0.12 | 1.83 | 15.92 | 0.027 |
|  | left-hippocampus | 0.42 | 2.44 | 2.37 | 0.12 | 1.47 | 16.12 | 0.040 |
|  | right-amygdala | 0.45 | 2.66 | 2.96 | 0.16 | 3.74 | 27.85 | 0.041 |
|  | right-hippocampus | 0.44 | 2.56 | 2.35 | 0.12 | 1.90 | 16.30 | 0.026 |

**Supplementary Table 8.** Diagnostic Performance of Plasma Biomarkers Across Clinical Subgroups

| **Subset** | **Biomarker** | **Cutoff** | **Acc** | **PPV** | **NPV** | **Sens** | **Spec** | **Acc_CI** | **PPV_CI** | **NPV_CI** | **Sens_CI** | **Spec_CI** | **Interm%** |
| --- | --- | --- | --- | --- | --- | --- | --- | --- | --- | --- | --- | --- | --- |
| CN | pT217_F | 0.13 | 0.74 | 0.24 | 0.9 | 0.45 | 0.78 | (0.68, 0.80) | (0.12, 0.35) | (0.86, 0.95) | (0.26, 0.64) | (0.72, 0.84) | 0.92 |
| CN | AB42_F | 35.15 | 0.84 | 0.2 | 0.87 | 0.07 | 0.96 | (0.79, 0.88) | (0.00, 0.50) | (0.83, 0.91) | (0.00, 0.17) | (0.93, 0.98) | 0.9 |
| CN | AB40_F | 300.99 | 0.62 | 0.17 | 0.88 | 0.46 | 0.64 | (0.55, 0.68) | (0.09, 0.25) | (0.83, 0.93) | (0.29, 0.63) | (0.57, 0.71) | 0.93 |
| CN | AB42_AB40_F | 0.1 | 0.8 | 0.2 | 0.88 | 0.17 | 0.9 | (0.75, 0.86) | (0.05, 0.38) | (0.83, 0.92) | (0.04, 0.32) | (0.86, 0.94) | 0.88 |
| CN | pT217_AB42_F | 0 | 0.68 | 0.18 | 0.89 | 0.42 | 0.72 | (0.62, 0.74) | (0.09, 0.29) | (0.84, 0.94) | (0.24, 0.62) | (0.65, 0.78) | 0.91 |
| CN | NfL_Q | 20.9 | 0.75 | 0.19 | 0.88 | 0.28 | 0.82 | (0.69, 0.80) | (0.07, 0.33) | (0.83, 0.93) | (0.11, 0.45) | (0.77, 0.87) | 0.92 |
| CN | GFAP_Q | 96.3 | 0.39 | 0.14 | 0.89 | 0.71 | 0.34 | (0.32, 0.45) | (0.08, 0.19) | (0.82, 0.96) | (0.54, 0.88) | (0.27, 0.41) | 0.9 |
| MCI | pT217_F | 0.44 | 0.77 | 0.48 | 0.84 | 0.44 | 0.86 | (0.69, 0.84) | (0.30, 0.67) | (0.76, 0.90) | (0.27, 0.62) | (0.80, 0.92) | 0.84 |
| MCI | AB42_F | 14.89 | 0.26 | 0.25 |  | 1 | 0.01 | (0.20, 0.33) | (0.19, 0.33) | (nan, nan) | (1.00, 1.00) | (0.00, 0.03) | 0.93 |
| MCI | AB40_F | 388.01 | 0.74 |  | 0.75 | 0.08 | 0.97 | (0.67, 0.81) | (nan, nan) | (0.68, 0.82) | (0.00, 0.18) | (0.94, 1.00) | 0.94 |
| MCI | AB42_AB40_F | 0.1 | 0.66 | 0.32 | 0.77 | 0.31 | 0.78 | (0.58, 0.73) | (0.18, 0.48) | (0.68, 0.84) | (0.17, 0.47) | (0.70, 0.85) | 0.89 |
| MCI | pT217_AB42_F | 0.02 | 0.8 | 0.56 | 0.85 | 0.45 | 0.9 | (0.73, 0.86) | (0.36, 0.76) | (0.78, 0.92) | (0.29, 0.64) | (0.83, 0.95) | 0.85 |
| MCI | NfL_Q | 13.4 | 0.38 | 0.27 | 0.78 | 0.82 | 0.23 | (0.31, 0.47) | (0.19, 0.36) | (0.62, 0.91) | (0.68, 0.93) | (0.15, 0.31) | 0.94 |
| MCI | GFAP_Q | 234.5 | 0.76 | 0.56 | 0.78 | 0.25 | 0.93 | (0.69, 0.83) | (0.31, 0.80) | (0.71, 0.85) | (0.12, 0.40) | (0.88, 0.97) | 0.89 |
| AD | pT217_F | 0.42 | 0.78 | 0.77 | 0.79 | 0.87 | 0.65 | (0.65, 0.90) | (0.61, 0.92) | (0.57, 1.00) | (0.73, 1.00) | (0.42, 0.86) | 0.7 |
| AD | AB42_F | 32.41 | 0.45 |  | 0.41 | 0.17 | 0.87 | (0.29, 0.61) | (nan, nan) | (0.24, 0.59) | (0.04, 0.33) | (0.69, 1.00) |  |
| AD | AB40_F | 223.73 | 0.6 | 0.58 |  | 1 | 0.06 | (0.45, 0.75) | (0.43, 0.74) | (nan, nan) | (1.00, 1.00) | (0.00, 0.19) |  |
| AD | AB42_AB40_F | 0.08 | 0.59 | 0.61 | 0.54 | 0.73 | 0.41 | (0.44, 0.74) | (0.42, 0.79) | (0.27, 0.82) | (0.52, 0.90) | (0.18, 0.63) | 0.87 |
| AD | pT217_AB42_F | 0.02 | 0.76 | 0.72 | 0.83 | 0.91 | 0.56 | (0.61, 0.88) | (0.56, 0.88) | (0.60, 1.00) | (0.78, 1.00) | (0.32, 0.79) |  |
| AD | NfL_Q | 33.3 | 0.59 | 0.87 | 0.52 | 0.32 | 0.94 | (0.41, 0.74) | (0.60, 1.00) | (0.33, 0.69) | (0.14, 0.52) | (0.81, 1.00) | 0.92 |
| AD | GFAP_Q | 148 | 0.61 | 0.6 |  | 0.9 | 0.24 | (0.45, 0.76) | (0.42, 0.76) | (nan, nan) | (0.75, 1.00) | (0.05, 0.46) |  |
| Total cohort | pT217_F | 0.39 | 0.81 | 0.58 | 0.85 | 0.42 | 0.92 | (0.77, 0.85) | (0.46, 0.70) | (0.81, 0.89) | (0.31, 0.53) | (0.89, 0.95) | 0.85 |
| Total cohort | AB42_F | 14.89 | 0.22 | 0.22 |  | 1 | 0 | (0.18, 0.26) | (0.18, 0.26) | (nan, nan) | (1.00, 1.00) | (0.00, 0.01) | 0.93 |
| Total cohort | AB40_F | 368.25 | 0.75 | 0.29 | 0.78 | 0.1 | 0.93 | (0.70, 0.79) | (0.14, 0.46) | (0.74, 0.83) | (0.04, 0.16) | (0.91, 0.96) | 0.93 |
| Total cohort | AB42_AB40_F | 0.1 | 0.74 | 0.25 | 0.79 | 0.1 | 0.92 | (0.69, 0.78) | (0.11, 0.38) | (0.74, 0.82) | (0.04, 0.17) | (0.89, 0.95) | 0.91 |
| Total cohort | pT217_AB42_F | 0.02 | 0.82 | 0.59 | 0.86 | 0.44 | 0.92 | (0.78, 0.86) | (0.47, 0.72) | (0.82, 0.90) | (0.33, 0.55) | (0.89, 0.95) | 0.84 |
| Total cohort | NfL_Q | 20.9 | 0.64 | 0.29 | 0.81 | 0.43 | 0.7 | (0.60, 0.69) | (0.21, 0.37) | (0.77, 0.86) | (0.33, 0.53) | (0.65, 0.75) | 0.91 |
| Total cohort | GFAP_Q | 195.8 | 0.72 | 0.34 | 0.82 | 0.35 | 0.82 | (0.68, 0.76) | (0.25, 0.44) | (0.78, 0.86) | (0.25, 0.46) | (0.77, 0.86) | 0.87 |

Diagnostic performance of seven biomarkers was evaluated in the total cohort as well as within Alzheimer’s disease (AD), cognitively normal (CN), and mild cognitive impairment (MCI) subgroups. Metrics included accuracy, sensitivity, specificity, positive predictive value (PPV), and negative predictive value (NPV). Full numeric results are presented in **Table 10**.

In the CN group, pT217_F (cutoff 0.13) had accuracy 0.74 (CI: 0.68–0.80), sensitivity 0.45 (CI: 0.26–0.64), specificity 0.78 (CI: 0.72–0.84), PPV = 0.24 (CI: 0.12–0.35), and NPV = 0.90 (CI: 0.86–0.95).
AB42_F (cutoff 35.15) yielded accuracy 0.84 (CI: 0.79–0.88), but sensitivity was very low (0.07, CI: 0.00–0.17), with specificity 0.96 (CI: 0.93–0.98), PPV = 0.20 (CI: 0.00–0.50), and NPV = 0.87 (CI: 0.83–0.91).
AB40_F (cutoff 366.32) gave accuracy 0.70 (CI: 0.64–0.76), sensitivity 0.13 (CI: 0.00–0.28), specificity 0.92 (CI: 0.88–0.96), PPV = 0.25 (CI: 0.05–0.45), and NPV = 0.83 (CI: 0.78–0.88).
The AB42/AB40 ratio (cutoff 0.10) showed accuracy 0.71 (CI: 0.65–0.77), sensitivity 0.13 (CI: 0.00–0.28), specificity 0.93 (CI: 0.89–0.97), PPV = 0.27 (CI: 0.06–0.47), and NPV = 0.83 (CI: 0.78–0.88).
pT217_AB42_F (cutoff 0.02) performed best, with accuracy 0.84 (CI: 0.79–0.88), sensitivity 0.47 (CI: 0.28–0.66), specificity 0.91 (CI: 0.87–0.95), PPV = 0.35 (CI: 0.20–0.50), and NPV = 0.88 (CI: 0.84–0.92).

In the MCI group, pT217_F (cutoff 0.44) yielded accuracy 0.77 (CI: 0.69–0.84), sensitivity 0.44 (CI: 0.27–0.62), specificity 0.86 (CI: 0.80–0.92), PPV = 0.48 (CI: 0.30–0.67), and NPV = 0.84 (CI: 0.76–0.90).
AB42_F (cutoff 14.89) reached accuracy 0.26 (CI: 0.20–0.33), perfect sensitivity (1.00, CI: 1.00–1.00), but near-null specificity (0.01, CI: 0.00–0.03). PPV was 0.25 (CI: 0.19–0.33), and NPV could not be defined.
AB40_F (cutoff 366.93) gave accuracy 0.72 (CI: 0.64–0.80), sensitivity 0.12 (CI: 0.00–0.25), specificity 0.92 (CI: 0.87–0.97), PPV = 0.38 (CI: 0.12–0.63), and NPV = 0.76 (CI: 0.69–0.83).
The AB42/AB40 ratio (cutoff 0.10) performed similarly, with accuracy 0.73 (CI: 0.65–0.81), sensitivity 0.12 (CI: 0.00–0.25), specificity 0.93 (CI: 0.88–0.97), PPV = 0.40 (CI: 0.13–0.67), and NPV = 0.76 (CI: 0.69–0.83).
pT217_AB42_F (cutoff 0.02) was the strongest in this group, achieving accuracy 0.80 (CI: 0.73–0.87), sensitivity 0.39 (CI: 0.22–0.57), specificity 0.90 (CI: 0.84–0.95), PPV = 0.52 (CI: 0.33–0.71), and NPV = 0.82 (CI: 0.75–0.89).

For the AD group, pT217_F (cutoff 0.42) yielded accuracy 0.78 (CI: 0.65–0.90), sensitivity 0.87 (CI: 0.73–1.00), specificity 0.65 (CI: 0.42–0.86), PPV = 0.77 (CI: 0.61–0.92), and NPV = 0.79 (CI: 0.57–1.00).
AB42_F (cutoff 32.41) was weaker, with accuracy 0.45 (CI: 0.29–0.61), sensitivity 0.17 (CI: 0.04–0.33), specificity 0.87 (CI: 0.69–1.00), undefined PPV, and NPV = 0.41 (CI: 0.24–0.59).
AB40_F (cutoff 292.24) showed accuracy 0.74 (CI: 0.59–0.88), sensitivity 0.13 (CI: 0.00–0.29), specificity 0.96 (CI: 0.88–1.00), PPV = 0.67 (CI: 0.19–1.00), and NPV = 0.73 (CI: 0.58–0.88).
The AB42/AB40 ratio (cutoff 0.08) achieved accuracy 0.72 (CI: 0.57–0.86), sensitivity 0.13 (CI: 0.00–0.29), specificity 0.96 (CI: 0.88–1.00), PPV = 0.67 (CI: 0.19–1.00), and NPV = 0.71 (CI: 0.56–0.86).
pT217_AB42_F (cutoff 0.02) demonstrated strong diagnostic balance, with accuracy 0.84 (CI: 0.72–0.95), sensitivity 0.80 (CI: 0.62–0.98), specificity 0.87 (CI: 0.69–1.00), PPV = 0.85 (CI: 0.69–1.00), and NPV = 0.82 (CI: 0.64–1.00).

In the total cohort, pT217_F with a cutoff of 0.39 achieved an accuracy of 0.81 (95% CI: 0.77–0.85), sensitivity of 0.42 (95% CI: 0.31–0.53), and specificity of 0.92 (95% CI: 0.89–0.95). PPV was 0.58 (95% CI: 0.46–0.70), and NPV was 0.85 (95% CI: 0.81–0.89).
AB42_F (cutoff 14.89) reached perfect sensitivity (1.00, CI: 1.00–1.00) but null specificity (0.00, CI: 0.00–0.01), producing low accuracy (0.22, CI: 0.18–0.26), PPV = 0.22 (CI: 0.18–0.26), and undefined NPV.
AB40_F (cutoff 368.25) showed accuracy of 0.75 (CI: 0.70–0.79), sensitivity 0.10 (CI: 0.04–0.16), and specificity 0.93 (CI: 0.91–0.96), with PPV = 0.29 (CI: 0.14–0.46) and NPV = 0.78 (CI: 0.74–0.83).
The AB42/AB40 ratio (cutoff 0.10) had accuracy 0.74 (CI: 0.69–0.78), sensitivity 0.10 (CI: 0.04–0.17), specificity 0.92 (CI: 0.89–0.95), PPV = 0.25 (CI: 0.11–0.38), and NPV = 0.79 (CI: 0.74–0.82).
pT217_AB42_F (cutoff 0.02) achieved the best balance, with accuracy 0.82 (CI: 0.78–0.86), sensitivity 0.44 (CI: 0.33–0.55), specificity 0.92 (CI: 0.89–0.95), PPV = 0.59 (CI: 0.47–0.72), and NPV = 0.86 (CI: 0.82–0.90).

**Associations Between Regional Tau PET SUVR and Cognitive Performance**

We examined associations between regional tau PET SUVR and cognitive performance (MMSE and CDR-SB) across diagnostic groups (CN, MCI, and AD) to evaluate the regional contribution of tau pathology to cognitive impairment.

**MMSE Associations**

In the AD group, MMSE scores showed significant associations with tau burden in ventricular and insular regions. Higher tau uptake in the third ventricle (r = 0.38, p = 0.018) and fourth ventricle (r = 0.39, p = 0.016) was associated with worse cognitive performance, while insular cortex tau was inversely related to MMSE (left insula: r = –0.38, p = 0.018; right insula: r = –0.37, p = 0.025), suggesting that both cortical and periventricular changes are linked to global cognitive decline in advanced disease.

In the MCI group, MMSE was most strongly associated with tau deposition in medial temporal and limbic regions, including the entorhinal cortex (ρ = –0.24 to –0.24, p ≤ 0.004), inferior parietal cortex (ρ = –0.22, p = 0.010), precuneus (ρ = –0.21, p = 0.013), posterior cingulate cortex (ρ = –0.20, p = 0.018), and amygdala (ρ = –0.18 to –0.22, p ≤ 0.030). Additional associations were observed in frontal and temporal association cortices, indicating early involvement of distributed cognitive networks. In CN participants, weaker but significant associations were observed primarily in temporal and limbic regions, including the entorhinal cortex (ρ ≈ 0.19–0.20, p < 0.01), fusiform gyrus, parahippocampal cortex, and frontal pole(Supplementary Table S11).

**CDR-SB Associations**

In the AD group, CDR-SB was strongly associated with tau burden in subcortical and striatal regions, including the caudate nucleus (r = 0.45–0.56, p < 0.01), putamen (r = 0.45–0.52, p < 0.01), accumbens area (r = 0.35, p = 0.035), and ventral diencephalic structures (r ≈ 0.33–0.33, p ≈ 0.04). In contrast, cerebellar cortex tau showed a negative association with CDR-SB (r = –0.36, p = 0.025), consistent with regionally specific disease effects rather than global tracer uptake artifacts.

In the MCI group, CDR-SB was predominantly associated with posterior cortical and occipital regions, including lingual gyrus (ρ = –0.21 to –0.27, p ≤ 0.01), pericalcarine cortex (ρ ≈ –0.21 to –0.22, p ≈ 0.009–0.012), and parietal regions including precuneus and inferior parietal cortex (ρ ≈ –0.18 to –0.22, p < 0.02). Additional associations were observed in anterior cingulate and cerebellar regions, suggesting that clinical progression in MCI is linked to both cortical and network-level tau accumulation (Supplementary Table S12).

**Supplementary Table 9**. Correlation between regional tau PET and MMSE across diagnostic groups

| Diagnosis | Regions | method | r | P value |
| --- | --- | --- | --- | --- |
| AD | ventricle 4th | pearson | 0.388 | 0.016 |
| AD | ctx insula | spearman | -0.382 | 0.018 |
| AD | ventricle 3rd | pearson | 0.381 | 0.018 |
| AD | ctx rh insula | spearman | -0.367 | 0.025 |
| CN | ctx lh entorhinal | spearman | 0.201 | 0.005 |
| CN | ctx entorhinal | spearman | 0.191 | 0.008 |
| CN | ctx rh frontalpole | spearman | 0.175 | 0.012 |
| CN | ctx rh parahippocampal | spearman | 0.180 | 0.012 |
| CN | ctx rh fusiform | spearman | 0.174 | 0.014 |
| CN | meta temporal | spearman | -0.167 | 0.017 |
| CN | ctx parahippocampal | spearman | 0.170 | 0.019 |
| CN | ctx fusiform | spearman | 0.155 | 0.029 |
| CN | ctx rh inferiortemporal | spearman | 0.153 | 0.031 |
| CN | ctx lh lateralorbitofrontal | spearman | 0.148 | 0.037 |
| CN | ctx insula | spearman | 0.143 | 0.043 |
| CN | ctx rh insula | spearman | 0.140 | 0.047 |
| MCI | ctx entorhinal | spearman | -0.242 | 0.003 |
| MCI | ctx lh entorhinal | spearman | -0.241 | 0.003 |
| MCI | left amygdala | spearman | -0.224 | 0.007 |
| MCI | ctx lh inferiorparietal | spearman | -0.224 | 0.010 |
| MCI | ctx rh entorhinal | spearman | -0.213 | 0.011 |
| MCI | ctx inferiorparietal | spearman | -0.218 | 0.011 |
| MCI | ctx lh precuneus | spearman | -0.215 | 0.013 |
| MCI | left accumbens area | spearman | -0.203 | 0.015 |
| MCI | ctx lh posteriorcingulate | spearman | -0.202 | 0.018 |
| MCI | left choroid plexus | spearman | 0.199 | 0.018 |
| MCI | ctx lh caudalmiddlefrontal | spearman | -0.199 | 0.022 |
| MCI | ctx lh supramarginal | spearman | -0.189 | 0.029 |
| MCI | amygdala | spearman | -0.182 | 0.030 |
| MCI | ctx lh parahippocampal | spearman | -0.175 | 0.040 |
| MCI | ctx rh inferiorparietal | spearman | -0.177 | 0.044 |
| MCI | ctx lh parsorbitalis | spearman | -0.168 | 0.047 |
| MCI | accumbens area | spearman | -0.164 | 0.049 |

**Supplementary Table 12**. Correlation between regional tau PET and CDR-SB across diagnostic groups

| Diagnosis | Regions | method | r | P value |
| --- | --- | --- | --- | --- |
| AD | right caudate | pearson | 0.565 | 0.000 |
| AD | caudate | pearson | 0.552 | 0.000 |
| AD | right putamen | pearson | 0.517 | 0.001 |
| AD | left caudate | pearson | 0.453 | 0.006 |
| AD | putamen | pearson | 0.451 | 0.006 |
| AD | right cerebellum cortex | pearson | -0.363 | 0.025 |
| AD | accumbens area | pearson | 0.348 | 0.035 |
| AD | left ventraldc | pearson | 0.335 | 0.040 |
| AD | right ventraldc | pearson | 0.335 | 0.040 |
| AD | ventraldc | pearson | 0.334 | 0.040 |
| AD | left putamen | pearson | 0.338 | 0.044 |
| MCI | ctx lh lingual | spearman | -0.266 | 0.002 |
| MCI | left choroid plexus | spearman | -0.219 | 0.009 |
| MCI | ctx pericalcarine | spearman | -0.219 | 0.009 |
| MCI | cerebellum cortex | spearman | -0.216 | 0.010 |
| MCI | left cerebellum cortex | spearman | -0.215 | 0.010 |
| MCI | ctx rh lingual | spearman | -0.224 | 0.011 |
| MCI | ctx rh pericalcarine | spearman | -0.212 | 0.011 |
| MCI | choroid plexus | spearman | -0.211 | 0.012 |
| MCI | right cerebellum cortex | spearman | -0.210 | 0.012 |
| MCI | ctx lh paracentral | spearman | -0.205 | 0.015 |
| MCI | ctx lingual | spearman | -0.208 | 0.018 |
| MCI | ctx lh bankssts | spearman | -0.200 | 0.020 |
| MCI | right choroid plexus | spearman | -0.189 | 0.024 |
| MCI | ctx lh pericalcarine | spearman | -0.183 | 0.030 |
| MCI | ctx lh caudal anterior cingulate | spearman | -0.183 | 0.032 |
| MCI | accumbens area | spearman | 0.172 | 0.037 |
| MCI | ctx rh caudal anterior cingulate | spearman | -0.174 | 0.042 |
| MCI | ctx isthmus cingulate | spearman | -0.175 | 0.047 |
| MCI | ctx rh rostral anterior cingulate | spearman | -0.167 | 0.050 |
